# Plasmalogen remodeling modulates macrophage response to cytotoxic oxysterols and atherosclerotic plaque vulnerability

**DOI:** 10.1016/j.xcrm.2025.102131

**Published:** 2025-05-08

**Authors:** Antoine Jalil, Thomas Pilot, Thibaut Bourgeois, Aline Laubriet, Xiaoxu Li, Marc Diedisheim, Valérie Deckert, Charlène Magnani, Naig Le Guern, Jean-Paul Pais de Barros, Maxime Nguyen, Gaëtan Pallot, Adrien Vouilloz, Lil Proukhnitzky, François Hermetet, Virginie Aires, Eric Lesniewska, Laurent Lagrost, Johan Auwerx, Wilfried Le Goff, Nicolas Venteclef, Eric Steinmetz, Charles Thomas, David Masson

**Affiliations:** 1 Université Bourgogne, UMR1231, 21000 Dijon, France; 2 INSERM, UMR1231, 21000 Dijon, France; 3 LipSTIC LabEx, 21000 Dijon, France; 4 CHRU Dijon Bourgogne, Department of Cardiovascular Surgery, Dijon University Medical Center, 21000 Dijon, France; 5 Laboratory of Integrative Systems Physiology, Institute of Bioengineering, École Polytechnique Fédérale de Lausanne, 1015 Lausanne, Switzerland; 6 Centre - Clinique Saint Gatien Alliance (NCT+), 37214 Saint-Cyr-sur-Loire, France; 7 Institut Necker-Enfants Malades, INSERM UMR-S1151, Université Paris Cité, 75015 Paris, France; 8 Lipidomic Analytic Platform, UBFC, 21000 Dijon, France; 9 CHRU Dijon Bourgogne, Department of Anesthesiology and Critical Care Medicine, Dijon University Medical Center, 21000 Dijon, France; 10 Laboratory of Physics, National Center for Scientific Research, URA 5027, UFR Sciences et techniques, 21000 Dijon, France; 11 Sorbonne Université, Inserm, ICAN Institut, UMR_S1166, Hôpital de la Pitié, 75013 Paris, France; 12 CHRU Dijon Bourgogne, Laboratory of Clinical Chemistry, 21000 Dijon, France

**Keywords:** macrophages, polyunsaturated FAs, ether lipids, atherosclerosis

## Abstract

Essential fatty acid metabolism in myeloid cells plays a critical but underexplored role in immune function. Here, we demonstrate that simultaneous inactivation of two key enzymes involved in macrophage polyunsaturated fatty acid (PUFA) metabolism—ELOVL5, which elongates long-chain PUFAs, and LPCAT3, which incorporates them into phospholipids—disrupts membrane organization by promoting the formation of cholesterol-enriched domains. This increases macrophage sensitivity to cytotoxic oxysterols and leads to more vulnerable atherosclerotic plaques with enlarged necrotic cores in a mouse model of atherosclerosis. In humans, analysis of 187 carotid plaques reveals a positive correlation between LPCAT3/ELOVL5-generated phospholipids—including arachidonate (C20:4 n-6)-containing ether lipids—and more stable plaque profiles. Additionally, Mendelian randomization analysis supports a causal relationship between *LPCAT3* expression and reduced risk of ischemic stroke. Our findings uncover a regulatory circuit essential for PUFA-containing phospholipid generation in macrophages, positioning PUFA-containing ether lipids as promising biomarkers and therapeutic targets.

## Highlights

• LPCAT3 and ELOVL5 coordinate PUFA metabolism in macrophages
• Dual LPCAT3/ELOVL5 deletion increases membrane cholesterol-enriched domains
• This alteration enhances macrophage sensitivity to cytotoxic oxysterols
• PUFA-containing ether lipids correlate with stable human atheroma plaques

## Graphical abstract

**Figure:**
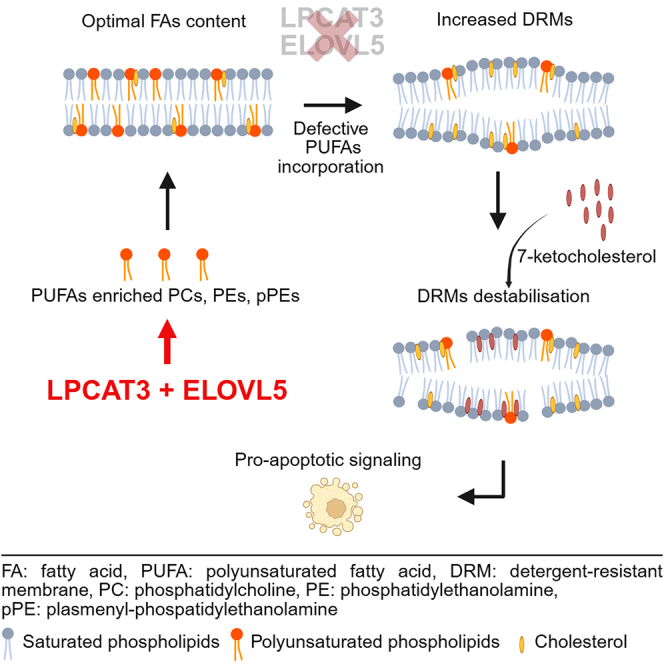

## Teaser

Jalil et al. reveal that macrophage phospholipid metabolism, regulated by key enzymes LPCAT3 and ELOVL5, influences cholesterol-enriched membrane domains and sensitivity to cytotoxic oxysterols. Their findings identify polyunsaturated fatty acid (PUFA)-containing ether lipids as potential biomarkers and therapeutic targets in atherosclerosis, linking PUFA-phospholipid metabolism to plaque stability and cardiovascular risk.

## Introduction

Macrophages are central in the onset of cardio-metabolic diseases such as atherosclerosis, obesity, type 2 diabetes and metabolic-associated steatohepatitis (MASH).^1^^,^^2^^,^^3^ Exhibiting significant cellular plasticity, macrophages adapt their activation status in response to a multitude of micro-environmental stimuli such as pathogens, apoptotic cells, lipoproteins, and cytokines.^3^ Of particular interest is the role of essential fatty acids (FAs) in modifying macrophage functions, an area that is increasingly gaining attention.^4^ Since FAs bearing double bonds at either the n-3 or the n-6 position cannot be synthesized by mammalian cells, macrophages are dependent on the general metabolic/dietary context as well as the local lipid environment for essential FA uptake. Furthermore, these essential FAs play an integral role in various aspects of macrophage biology. Indeed, unsaturated FAs act as precursors of bioactive molecules produced by macrophages including prostaglandins, leukotrienes, and specialized pro-resolving mediators.^5^^,^^6^

As components of glycerophospholipids, polyunsaturated FAs (PUFAs) have a pivotal role in membrane biophysics and modulate membrane-associated cellular processes such as fluidity, curvature, and micro domain formation as well as their susceptibility to peroxidation.^7^^,^^8^^,^^9^

Even though dietary supplementation with essential FAs has been shown to directly affect the FA composition of macrophages,^10^^,^^11^ it appears crucial to consider that myeloid cells have the ability to metabolize these essential FAs supplied by their environment. This metabolism can lead toward either catabolic pathways or the synthesis of secondary FAs through activation, desaturation, and elongation reactions.^12^ Another aspect is the poor understanding of how regulatory pathways drive the specific addressing of these PUFAs into phospholipids (PLs) or other lipid molecules such as cholesteryl esters (CEs).

Recent studies targeting saturated FAs synthesis or sphingolipid metabolism have highlighted the potential of lipid anabolic pathways to control membrane organization and inflammatory processes in macrophages.^13^^,^^14^ However, very few studies have sought to target specifically the pathways leading to the PUFA incorporation into cellular lipids within macrophages and to assess the consequences on the composition and organization of cellular membranes.

Our team and other groups have recently been interested in the lysophosphatidylcholine acyltransferase 3 (LPCAT3), a member of the lysophospholipid acyltransferase (LPLAT) family. LPLATs, involved in the Lands cycle, are able to promote the transfer of an acyl-coenzyme A (AcylCoA) toward the sn-2 position of a lysophospholipid, leading to the synthesis of a diacylglycerophospholipid.^15^^,^^16^^,^^17^ In macrophages, LPCAT3 is a key determinant of arachidonic acid (AA) and eicosapentaenoic acid (EPA) metabolism, and *Lpcat3*^−/−^ macrophages display marked reductions in the AA and EPA contents of diacylphospholipids.^18^ Interestingly, this defective incorporation of AA within PLs was associated with a significant accumulation of its elongation product, namely adrenic acid (ADA, C22:4 n-6), in PLs, CEs, and free FA. While this elongated FA exerts distinct biological actions on Liver X receptor (LXR),^18^ it could also signify a compensatory mechanism employed by macrophages to uphold cellular PUFA homeostasis. Given the importance of maintaining PUFA homeostasis, we hypothesized that this process could involve redundant pathways, including those responsible for FA elongation. Consequently, a “one hit” strategy may not be sufficient to induce major alterations in membrane PUFA homeostasis. To test this hypothesis, we screened for enzymes involved in FA elongation to identify potential compensatory mechanisms maintaining PUFA incorporation in response to LPCAT3 deficiency. We interrogated whether simultaneously targeting both pathways would significantly alter the PUFA content of macrophages and whether it could affect the development of atherosclerosis.

In the present study, we determine that the elongation of very long-chain fatty acids protein 5 (ELOVL5) is involved in the elongation of AA in ADA in *Lpcat3*-deficient macrophages.

In accordance with our hypothesis, a combined *Lpcat3/Elovl5* deletion significantly reduces the PUFA content of the PLs, leading to major alterations in membrane cholesterol distribution and cholesterol-enriched domain formation. We show in an atherosclerosis mouse model that a combined *Lpcat3/Elovl5* deletion in macrophages results in higher necrotic core area, a phenomenon linked to heightened macrophage sensitivity to cytotoxic oxysterols such as 7-ketocholesterol (7-KC). Finally, we characterized in human carotid plaques from patients having undergone endarterectomy that PLs generated by the LPCAT3-ELOVL5 axis, and more specifically AA- or ADA-containing ether lipids, correlate with a more favorable atheroma plaque profile.

## Results

### Complementary roles of Lpcat3 and Elovl5 in modulating PUFA content of PLs in macrophages

**Figure 1:**
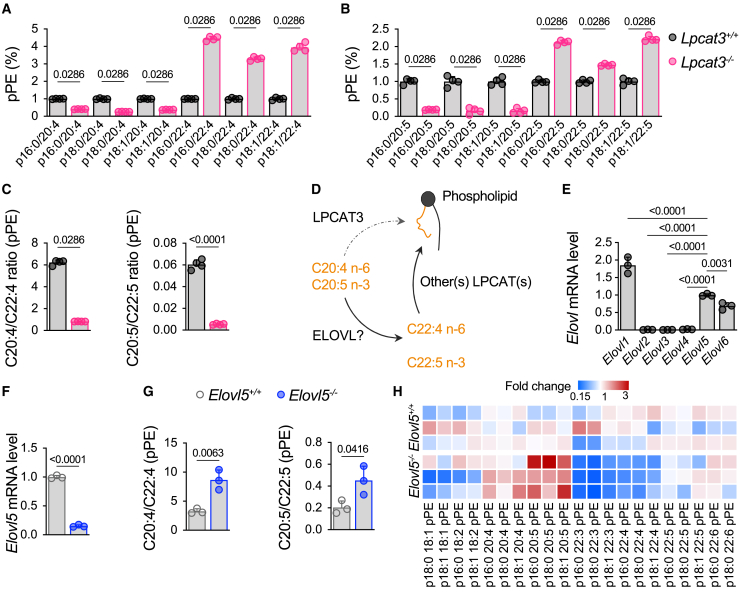
Distinct roles of Elovl5 and Lpcat3 in PL remodeling in murine macrophages (A and B) Amounts of pPEs containing C20:4 n-6, C22:4 n-6, C20:5 n-3, and C22:5 n-3 in *Lpcat3*^*+/+*^ and *Lpcat3*^−/−^ primary macrophages. Data are expressed as percentage of total pPEs and are normalized as 1 in the *Lpcat3*^*+/+*^ group (*n* = 4). (C) Ratio of C20:4/C22:4 n-6 and ratio of C20:5/C22:5 n-3 in pPEs (*n* = 4). (D) Graphical illustration of the compensatory system triggered by *Lpcat3* deficiency. (E) mRNA levels of the *Elovl* family members in wild-type (WT) primary bone marrow-derived macrophages normalized as 1 against *Elovl5* expression (*n* = 3). (F) *Elovl5* relative mRNA levels in CRISPR-Cas9 modified RAW 264.7 macrophages normalized as 1 in the *Elovl5*^*+/+*^ group (*n* = 3). (G) Ratio of C20:4/C22:4 n-6 and ratio of C20:5/C22:5 n-3 in pPEs in *Elovl5*^*+/+*^ and *Elovl5*^−/−^ cells. (H) Heatmap of pPEs in *Elovl5* CRISPR-Cas9 modified RAW 264.7 macrophages. Data are expressed as percentage of total phospholipids subclasses and are normalized as 1 in the *Elovl5*^*+/+*^ group (*n* = 3). Values are mean ± SD. Statistical analysis was performed with unpaired t test or Mann-Whitney. See also Figure S1.

Consistent with earlier works including ours,^18^^,^^19^^,^^20^^,^^21^ we confirmed that *Lpcat3*^−/−^ macrophages derived from fetal livers of E14 mouse embryos had a major decrease in AA and EPA within plasmalogens as compared to control cells (Figures 1A and 1B). As evidenced here, these decreases in C20 PUFAs are associated with a compensatory increase in the relative amounts of C22 PUFAs, such as C22:4 n-6 (ADA) and C22:5 n-3 (docosapentaenoic acid) as exemplified within the plasmalogen family (Figure 1C). Strikingly, the increase in C22 PUFAs triggered by the *Lpcat3* deletion appears to fully compensate the reductions in AA and EPA leading in fine to the conservation of an analogous PUFA content within PLs (Figures 1A and 1B). Similar changes are also observed with phosphatidylethanolamines (PEs) (Figure S1A) and phosphatidylcholines (PCs) (Figure S1B). Altogether, our findings suggest that, while LPCAT3 is required for the incorporation of C20 PUFAs into PLs, in *Lpcat3* deficient cells these FAs are efficiently converted into C22 PUFAs by an elongase-type enzyme and incorporated into PLs by LPCAT3-independent mechanisms (Figure 1D).

It has been documented that two members of the elongation of very long-chain protein family (ELOVL), namely ELOVL2 and ELOVL5, are the primary elongases needed for PUFA synthesis.^22^ We evaluated in an unbiased manner the basal expression of all *Elovl* genes in bone marrow-derived macrophages (BMDMs) and found nearly undetectable mRNA levels for *Elovl2* compared to the relatively high expression of *Elovl5* (Figure 1E). This prompted us to generate RAW 264.7 cells deficient for *Elovl5* (*Elovl5*^*/−*^) using a CRISPR-Cas9 approach (Figure 1F), and we observed a marked increase in C20:4/C22:4 n-6 and C20:5/C22:5 n-3 ratios in plasmenyl-ethanolamines compared to control cells (Figures 1G and 1H). Again, similar decreases in C22 PUFAs are also observed in PEs (Figure S1C) and PCs (Figure S1D) Therefore, ELOVL5 emerged as the critical player involved in the elongation of C20 to C22 PUFAs in macrophages, and this could compensate for *Lpcat3* deficiency (Figure 1D).

### *Lpcat3/Elovl5* deficiency alters PUFA content of PLs in macrophages

**Figure 2:**
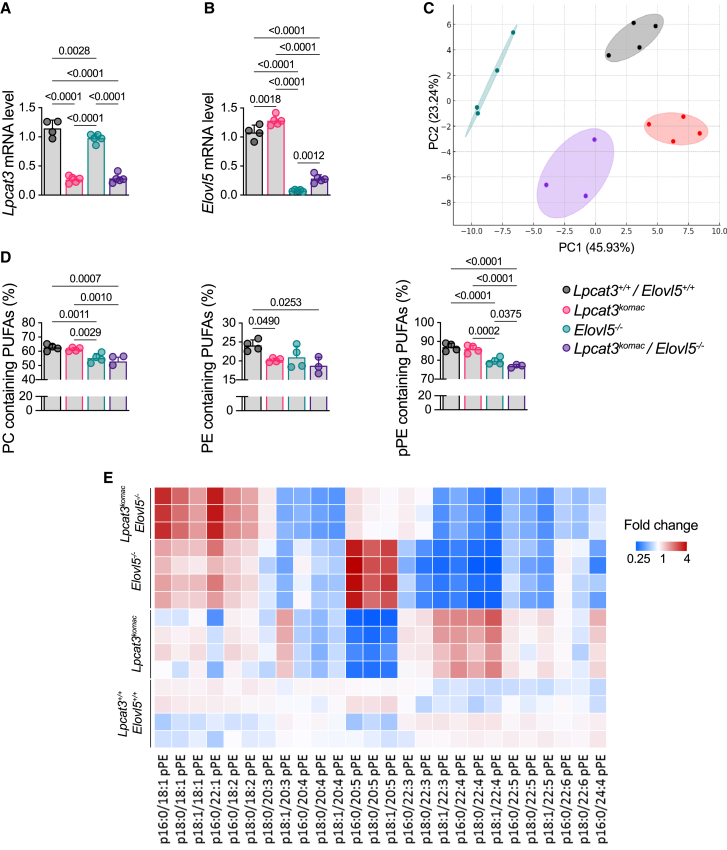
Combined Lpcat3/Elovl5 deficiency synergistically alters PUFA membrane composition in mouse primary macrophages (A and B) *Lpcat3* and *Elovl5* relative mRNA levels in *Lpcat3*^*+/+*^/*Elovl5*^*+/+*^, *Lpcat3*^*Komac*^, *Elovl5*^−/−^, and *Lpcat3*^*Komac*^/*Elovl5*^−/−^ primary macrophages normalized as 1 in the *Lpcat3*^*+/+*^/*Elovl5*^*+/+*^ group (*n* = 4). (C) Principal-component analysis of phospholipid species in *Lpcat3*^*+/+*^/*Elovl5*^*+/+*^, *Lpcat3*^*Komac*^, *Elovl5*^−/−^, and *Lpcat3*^*Komac*^/*Elovl5*^−/−^ primary macrophages. (D) Percentage of pPEs, PCs, and PEs containing PUFAs in each subclass in *Lpcat3*^*+/+*^/*Elovl5*^*+/+*^, *Lpcat3*^*Komac*^, *Elovl5*^−/−^, and *Lpcat3*^*Komac*^/*Elovl5*^−/−^ primary macrophages. (E) Heatmap of pPEs in *Lpcat3*^*+/+*^/*Elovl5*^*+/+*^, *Lpcat3*^*Komac*^, *Elovl5*^−/−^, and *Lpcat3*^*Komac*^/*Elovl5*^−/−^ primary macrophages. Data are expressed as percentage of total phospholipid subclass (*Lpcat3*^*+/+*^/*Elovl5*^*+/+*^, *Lpcat3*^*Komac*^, *Elovl5*^−/−^: *n* = 4; *Lpcat3*^*Komac*^/*Elovl5*^−/−^: *n* = 3). Values are mean ± SD. Statistical analysis was performed with one-way ANOVA (4 or more groups). See also Figures S2 and S3.

To directly test our hypothesis, we established a double *Lpcat3/Elovl5*-deficient mouse model by cross-breeding mice deficient for *Lpcat3* in myeloid cells, using a LysM^Cre^ strategy (*Lpcat3*^*Komac*^ mice) with *Elovl5*-deficient mice (*Elovl5*^−/−^) (Figures S2A and S2B). Knockdown efficiency was validated by an 80% decrease in *Lpcat3* mRNA levels in both *Lpcat3*^*Komac*^ and *Lpcat3*^*Komac*^*/Elovl5*^−/−^ BMDMs (Figure 2A). The same decrease was observed in *Elovl5* expression in *Elovl5*^−/−^ and *Lpcat3*^*Komac*^*/Elovl5*^−/−^macrophages compared to their control counterparts (Figure 2B).

Thus, we performed a comprehensive lipidomic analysis of diacylglycerophospholipid species including PEs and PCs, as well as plasmalogens, in these cells. A principal-component analysis (PCA) of these PLs showed a strong genotype clustering in the first two dimensions (Figure 2C). Similar to *Lpcat3*^*Komac*^ macrophages, *Lpcat3*^*Komac*^*/Elovl5*^−/−^ macrophages exhibited a decrease in diacylglycerophospholipids and plasmalogens containing AA or EPA (Figures 2D, 2E, and S3A). Likewise, both *Elovl5*^−/−^ and *Lpcat3*^*Komac*^*/Elovl5*^−/−^ macrophages showed a decrease in PLs containing C22 PUFAs (Figures 2D, 2E, and S3A). In *Lpcat3*^*Komac*^*/Elovl5*^−/−^ macrophages, the decrease in C20 PUFAs was observed without a compensatory increase in PLs containing C22 PUFAs thus confirming our primary hypothesis (Figures 2D and 2E). These macrophages, along with *Elovl5*^*Ko*^ macrophages, displayed an enrichment in PL species containing saturated or monounsaturated FAs (MUFAs) with shorter carbon chain lengths, which was more pronounced in *Lpcat3*^*Komac*^*/Elovl5*^−/−^ macrophages (Figures 2E and S3A). This shift toward MUFAs observed in *Lpcat3*^*Komac*^*/Elovl5*^−/−^ macrophages was linked to the induction of FA desaturases, including Stearoyl-CoA Desaturase 1 (*Scd1*) and Stearoyl-CoA Desaturase 2 (*Scd2)* (Figure S3B). Overall, the main consequence of the double *Lpcat3/Elovl5* deletion was a decrease in the percentage of PL species (plasmalogens, PC, and PE) containing PUFAs.

### Lpcat3/Elovl5 deficiency leads to an increase in cholesterol-enriched domains within plasma membrane

Alteration of the PUFA content of PLs has been previously shown to alter cellular membrane properties and microdomain formation. This is due to the higher affinity of cholesterol and sphingolipids for saturated FAs and MUFAs compared to PUFAs.^23^^,^^24^

**Figure 3:**
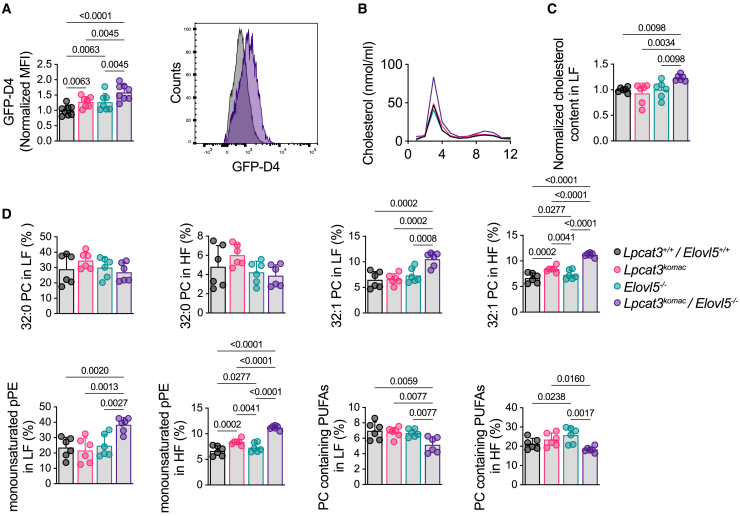
Combined Lpcat3 and Elovl5 deficiency affects cholesterol distribution within plasma membrane (A) Quantification of cholesterol-enriched domains in the plasma membrane by flow cytometry with GFP-D4 probe in *Lpcat3*^*+/+*^/*Elovl5*^*+/+*^, *Lpcat3*^*Komac*^, *Elovl5*^−/−^, and *Lpcat3*^*Komac*^/*Elovl5*^−/−^ primary macrophages. Data are expressed as mean fluorescent intensity and are normalized as 1 in the *Lpcat3*^*+/+*^/*Elovl5*^*+/+*^ group (*Lpcat3*^*+/+*^/*Elovl5*^*+/+*^, *Lpcat3*^*Komac*^/*Elovl5*^−/−^: *n* = 8 *Lpcat3*^*Komac*^, *Elovl5*^−/−^: *n* = 7). (B) Cholesterol distribution in fractions obtained by sucrose gradient ultracentrifugation in *Lpcat3*^*+/+*^/*Elovl5*^*+/+*^, *Lpcat3*^*Komac*^, *Elovl5*^−/−^, and *Lpcat3*^*Komac*^/*Elovl5*^−/−^ primary macrophages (*n* = 6). (C) Cholesterol content in light fractions (3–5). Data are normalized at 1 in the *Lpcat3*^*+/+*^/*Elovl5*^*+/+*^ group (*n* = 6). (D) Percentage of PCs containing 32:1 PC, 32:0 PC, PUFAs, and pPEs containing MUFAs in heavy and light fractions. Data are expressed as percentage of total PCs or total pPEs (*n* = 6). Values are mean ± SD. Statistical analysis was performed with one-way ANOVA. See also Figure S4.

The quantification of cholesterol-enriched domains within plasma membrane was performed by using the D4 fragment of perfringolysin O fused to a GFP fluorochrome.^25^ Flow cytometry analysis revealed an increase in the fluorescent intensity in *Lpcat3*^*Komac*^/*Elovl5*^−/−^ macrophages as compared to other genotypes (Figure 3A), reflecting a potential cholesterol redistribution within the plasma membrane leading to increased cholesterol-enriched domains. This prompted us to investigate the structure and the lipid composition of these particular micro-domains. Detergent-resistant membranes (DRMs) were isolated from BMDMs. As expected, detergent-resistant fractions (light fractions) corresponded to fractions 3 and 4, which contained the highest level of cholesterol (Figure 3B). Thus, we confirmed a significant increase in cholesterol level within DRMs in *Lpcat3*^*Komac*^/*Elovl5*^−/−^ macrophages as compared to other genotypes (Figure 3C). No changes for cholesterol were observed in the heavy fractions (Figure S4A).

Analysis of major PCs, PEs, and plasmenyl-PE (pPE) species between light and heavy fractions was also performed. Qualitatively, light fractions were enriched with lipids containing saturated FA or MUFA chains such as 32:0 PC (4.8% of PCs in heavy fractions vs. 29.0% in the light fractions) (Figure 3D). Notably, the *Lpcat3*^*Komac*^/*Elovl5*^−/−^ macrophages displayed a distinct lipid signature as compared to the three other genotypes with an increase in PC, PE, and pPE containing MUFAs in both light or heavy fractions as well as a reduced PUFA content (Figures 3D and S4B). As shown in Figure S4C, PCA showed that *Lpcat3*^*Komac*^/*Elovl5*^−/−^ macrophages differ from the other genotypes according to the PC2 dimension, with PC and pPE containing MUFAs being the major contributors.

### Combined bone marrow Lpcat3/Elovl5 deficiency promotes necrotic core formation in Ldlr^−/−^ recipient mice

We investigated whether an alteration of the PUFA homeostasis triggered by a combined *Lpcat3/Elovl5* deficiency in macrophages would affect the atherosclerotic process in a mouse model of hypercholesterolemia. Hence, recipient *Ldlr*^−/−^ mice were lethally irradiated before transplantation with hematopoietic cells collected from *Lpcat3*^*+/+*^/*Elovl5*^*+/+*^, *Lpcat3*^*Komac*^, *Elovl5*^−/−^, and *Lpcat3*^*Komac*^/*Elovl5*^−/−^ bone marrows. After four weeks of recovery, mice were fed with a western-type diet for twelve weeks before sacrifice and atherosclerotic lesion evaluation. The four groups did not display significant variations in their hematological parameters (Figure S5A) or peripheral blood monocyte subset count (Figure S5B). Analysis of plasma lipids did not reveal any difference in total or high-density lipoprotein cholesterol levels. However, there was a decrease in plasma triglycerides levels in the *Lpcat3*^*Komac*^/*Elovl5*^−/−^ group (Figures S5C and S5D).

**Figure 4:**
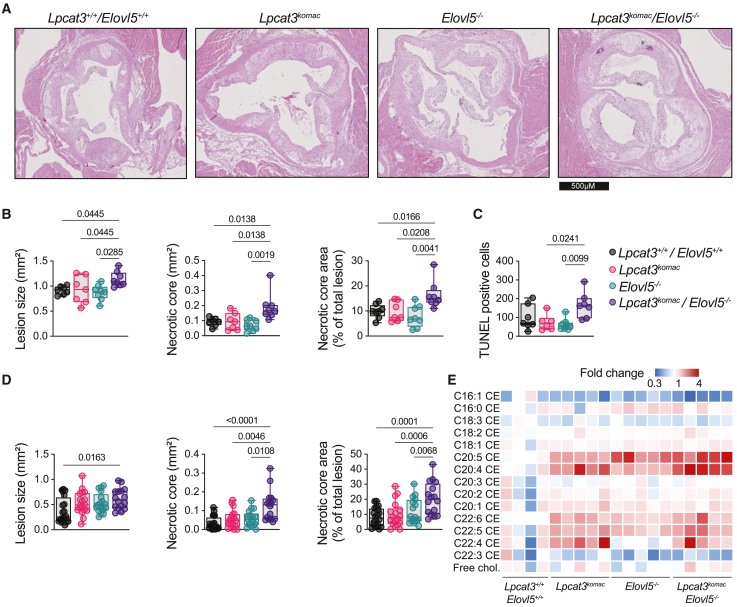
Combined *Lpcat3*/*Elovl5* deficiency in macrophages promotes necrotic core formation in *Ldlr*^***−/−***^ recipient mice (A) Representative images of aortic valve lesions in female Ldlr^−/−^ mice transplanted with *Lpcat3*^*+/+*^/*Elovl5*^*+/+*^, *Lpcat3*^*Komac*^, *Elovl5*^−/−^, and *Lpcat3*^*Komac*^/*Elovl5*^−/−^ hematopoietic cells. (B) Atherosclerotic lesion size (mm^2^) and necrotic core area (mm^2^ and percentage of total lesion area) quantification in female *Ldlr*^−/−^ mice transplanted with *Lpcat3*^*+/+*^/*Elovl5*^*+/+*^, *Lpcat3*^*Komac*^, *Elovl5*^−/−^, and *Lpcat3*^*Komac*^/*Elovl5*^−/−^ hematopoietic cells (*Lpcat3*^*+/+*^/*Elovl5*^*+/+*^, *Lpcat3*^*Komac*^*n* = 7, *Elovl5*^−/−^, *Lpcat3*^*Komac*^/*Elovl5*^−/−^*n* = 8). (C) Quantification by TUNEL of apoptotic cells within atheroma lesions in female *Ldlr*^−/−^ mice transplanted with *Lpcat3*^*+/+*^/*Elovl5*^*+/+*^, *Lpcat3*^*Komac*^, *Elovl5*^−/−^, and *Lpcat3*^*Komac*^/*Elovl5*^−/−^ hematopoietic cells (*Lpcat3*^*+/+*^/*Elovl5*^*+/+*^, *Lpcat3*^*Komac*^*n* = 7, *Elovl5*^−/−^, *Lpcat3*^*Komac*^/*Elovl5*^−/−^*n* = 8). (D) Atherosclerotic lesion size (mm^2^) and necrotic core area (mm^2^ and percentage of total lesion area) quantification in male *Ldlr*^−/−^ mice transplanted with *Lpcat3*^*+/+*^/*Elovl5*^*+/+*^, *Lpcat3*^*Komac*^, *Elovl5*^−/−^, and *Lpcat3*^*Komac*^/*Elovl5*^−/−^ hematopoietic cells (*Lpcat3*^*+/+*^/*Elovl5*^*+/+*^*n = 20*, *Lpcat3*^*Komac*^*n* = 18, *Elovl5*^−/−^*n* = 17, *Lpcat3*^*Komac*^/*Elovl5*^−/−^*n* = 16). (E) Heatmap of cholesteryl ester in the atheroma plaque from male *Ldlr*^−/−^ mice transplanted with *Lpcat3*^*+/+*^/*Elovl5*^*+/+*^, *Lpcat3*^*Komac*^, *Elovl5*^−/−^, and *Lpcat3*^*Komac*^/*Elovl5*^−/−^ hematopoietic cells (*Lpcat3*^*+/+*^/*Elovl5*^*+/+*^*n = 4*, *Lpcat3*^*Komac*^*n* = 5, *Elovl5*^−/−^*n* = 5, *Lpcat3*^*Komac*^/*Elovl5*^−/−^*n* = 5). Data are expressed in percentage of total CE subclass and normalized at 1 in the *Lpcat3*^*+/+*^/*Elovl5*^*+/+*^ group. Medians are indicated with the black horizontal bar. Error bars represent min. to max. Statistical analysis was performed with one-way ANOVA or Kruskal-Wallis. See also Figure S5.

Analysis of atherosclerotic lesions after twelve weeks of diet revealed that, in contrast to single knockout, a significant increase in lesion sizes within aortic roots was observed in female *Ldlr*^−/−^ recipient mice transplanted with *Lpcat3*^*Komac*^/*Elovl5*^−/−^ hematopoietic cells (Figures 4A and 4B). Importantly, *Ldlr*^−/−^ transplanted with *Lpcat3*^*Komac*^/*Elovl5*^−/−^ hematopoietic cells developed significantly more necrotic areas as compared to all other groups (Figure 4B). In accordance, TUNEL staining revealed an increase in apoptotic cell accumulation in *Ldlr*^−/−^ transplanted with *Lpcat3*^*Komac*^/*Elovl5*^−/−^ hematopoietic cells as compared to single knockouts (Figure 4C). Similar observations were made in male recipient *Ldlr*^−/−^ mice with a small but significant increase in lesion area in the *Lpcat3*^*Komac*^/*Elovl5*^−/−^ group as compared to the *Lpcat3*^*+/+*^/*Elovl5*^*+/+*^ group and a much more pronounced increase in necrotic area in the *Lpcat3*^*Komac*^/*Elovl5*^−/−^ group as compared to the other genotypes (Figure 4D).

Analysis of single-cell RNA sequencing (RNA-seq) datasets publicly available highlighted that *Lpcat3* and *Elovl5* are co-expressed in Trem2+ foamy macrophages within the plaque^26^ (Figure S5E) suggesting they might potentially affect PUFA metabolism directly within the atheroma plaque. Indeed, by analyzing the CEs from mouse aortic plaques, we observed a marked increase in AA and ADA within CEs in *Ldlr*^−/−^ mice transplanted with *Lpcat3*^*Komac*^ hematopoietic cells, which was more pronounced in the mice that received *Lpcat3*^*Komac*^/*Elovl5*^−/−^ hematopoietic cells (Figure 4E). These changes mirrored the defective incorporation of PUFAs in PLs; therefore, there was a redistribution of PUFAs from pPE to CEs (Figure S5F). As shown in Figure S5G, PCA analysis showed that *Lpcat3*^*Komac*^/*Elovl5*^−/−^ macrophages differ from the other genotypes according to the principal component 1 (PC1) and principal component 2 (PC2) dimension, with PC containing MUFAs and PEs containing 20:4 FAs being the major contributors.

### *Lpcat3*^*Komac*^*/Elovl5*^−/−^ macrophages display an increased sensitivity to 7-KC that is rescued by plasmalogen delivery

**Figure 5:**
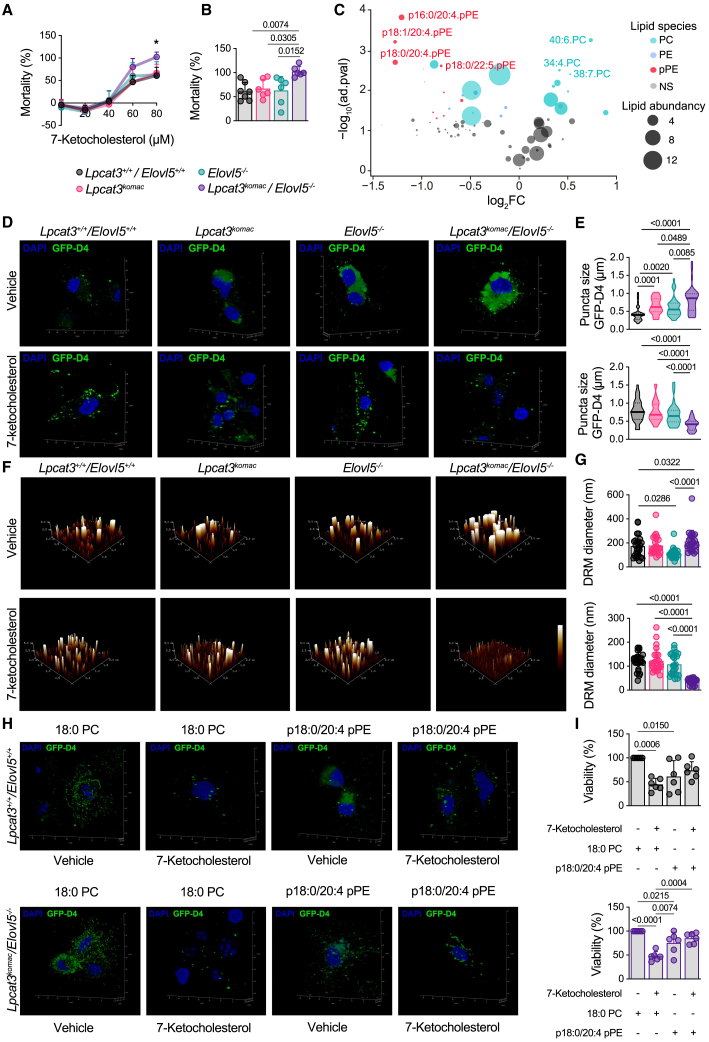
Lpcat3 and Elovl5 modulate the sensitivity of macrophages to cytotoxic oxysterols through DRM remodeling (A and B) Impact of 7-ketocholesterol treatment on macrophage mortality with lactate deshydrogenase (LDH) assay after 18 h of treatment (0–80 μM) (*n* = 7). (C) Volcano plot of major lipid species significantly depleted or enriched after 7-ketocholesterol treatment in *Lpcat3*^*+/+*^/*Elovl5*^*+/+*^ primary macrophages. Data are expressed in fold change compared to untreated condition. Bubble size represents lipid abundancy (*n* = 4). (D and E) Analysis of cholesterol-enriched domains by confocal microscopy using the GFP-D4 probe in macrophages treated or not with 7-ketocholesterol in *Lpcat3*^*+/+*^/*Elovl5*^*+/+*^, *Lpcat3*^*Komac*^, *Elovl5*^−/−^, and *Lpcat3*^*Komac*^/*Elovl5*^−/−^ primary macrophages. Puncta size was measured with LAS X and ImageJ software. Images are representatives of 4 experiments (*n* = 35). (F and G) Analysis of DRMs by atomic force microscopy using light fractions separated by ultracentrifugation from macrophages treated or not with 7-ketocholesterol. Images were analyzed with NanoScope Analysis 2.0. Images are representative of 4 experiments (*n* = 20). (H and I) Impact of 18:0 PC or p18:0/20:4 pPE supplementation on viability and cholesterol-enriched domain formation assessed by confocal microscopy and LDH assay. Data are representative of 3 experiments (*n* = 6). Values are mean ± SD. Statistical analysis was performed with one-way ANOVA or Kruskal-Wallis or Student’s t test for volcano plot with adjusted *p* values. See also Figures S6 and S7.

In order to delve deeper into the potentially involved mechanism(s), and particularly to test the hypothesis of higher macrophage mortality within atheroma plaques, we assessed *in vitro* the susceptibility of macrophages to a cytotoxic oxysterol found abundantly in atherosclerotic plaques, namely 7-KC.^27^ The cytotoxicity of oxysterols, and particularly that of 7-KC, is linked to their accumulation in cholesterol-enriched domains.^28^^,^^29^ Hence, we postulated that the variation in DRM composition observed within the different genotypes could influence the vulnerability of macrophages to cytotoxic oxysterols. As shown in Figure 5A, macrophages from *Lpcat3*^*Komac*^*/Elovl5*^−/−^ mice exhibited an increased sensitivity to 7-KC treatment as compared to the other genotypes, particularly at 80 μM (Figure 5B). Furthermore, targeted PL analysis revealed that 7-KC induced a selective depletion of plasmalogens containing AA (Figure 5C), these species being precisely among the most modulated by the LPCAT3-ELOVL5 axis. Analysis of DRMs, by the GFP-D4 probe (Figures 5D and 5E) or by atomic force microscopy (Figures 5F and 5G), showed a more pronounced effect in *Lpcat3*^*Komac*^*/Elovl5*^−/−^ macrophages, with an almost complete disappearance of these domains in 7-KC-treated BMDMs.

To assess whether AA-containing plasmalogens could directly exert a protective effect against 7-KC, macrophages were incubated for 24 h with liposomes enriched with p18:0/20:4 pPE or with control liposomes (18:0 PC) prior to 7-KC treatment (Figures S6A and S6B). As shown in Figure 5H, incubation with plasmalogen-enriched liposomes limits the impact of 7-KC on the marking of cholesterol-enriched domains in *Lpcat3*^*Komac*^*/Elovl5*^−/−^ macrophages (Figures 5H and S6C). Furthermore, while in the *Lpcat3*^*+/+*^/*Elovl5*^*+/+*^ macrophages plasmalogen-enriched liposomes induce cytotoxicity, possibly due to an overload of AA-containing plasmalogens (Figure 5I), they almost abolished completely the mortality induced by 7-KC in *Lpcat3*^*Komac*^*/Elovl5*^−/−^ macrophages (Figures 5I and S6D).

Finally, we performed another rescue experiment by treating BMDMs with GW3965, an LXR agonist. Both LPCAT3 and ELOVL5 are described as LXR targets, even though ELOVL5 is not directly induced in murine macrophages.^20^^,^^30^ Furthermore, LXR-dependent pathways (ABCG1) are known to protect macrophages against oxysterol toxicity.^31^

As expected, GW3965 treatment significantly increased the levels of LXR targets such as Abca1, Abcg1, and Lpcat3 in macrophages (Figure S6E) and provided notable protection against 7-KC-induced cell death (Figure S6F). Interestingly, while *Lpcat3*^*Komac*^*/Elovl5*^−/−^ macrophages exhibited a trend toward protection, the effect of LXR agonist pretreatment was less effective in preventing 7-KC-induced mortality in these cells (Figure S6F).

### Exploring *LPCAT3* and *ELOVL5* expression and potential role in human plaques through single-cell RNA-seq and Mendelian randomization

**Figure 6:**
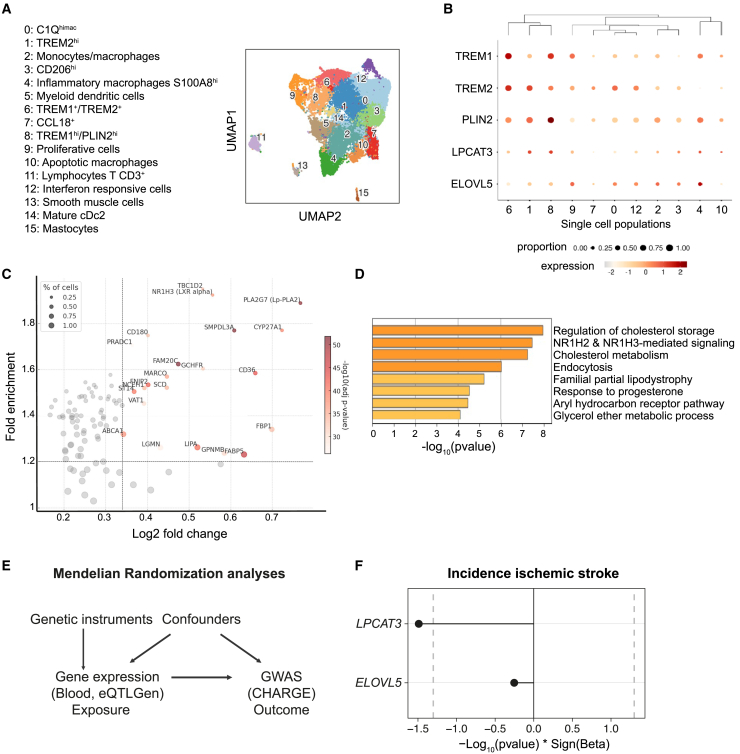
Analysis of LPCAT3 and ELOVL5 expression and potential role in human plaques through single-cell RNA sequencing and Mendelian randomization (A) UMAP visualization of myeloid-cell clusters in human carotid plaques (clusters labeled 0–15, annotations shown on the left). (B) Bubble plot showing expression levels and proportion of cells expressing specific genes across clusters. Dot size indicates the proportion of cells, and color intensity represents expression level. (C) Scatterplot displaying fold enrichment (percentage of cells expressing the gene in LPCAT3+/percentage of cells expressing the gene in all myeloid cells) vs. log2 fold change for gene expression in LPCAT3+ macrophages, with dot size corresponding to the percentage of cells expressing each gene. (D) Bar plot of enriched pathways and biological processes in LPCAT3-positive macrophages, with significance represented by −log10(p). (E) Schematic of Mendelian randomization analysis. (F) The effects of whole blood *ELOVL5* and *LPCAT3* gene expression on the incidence of ischemic stroke.

To more directly evaluate the relevance of our observations in humans, we recently analyzed published single-cell RNA-seq data from human carotid plaques^32^ and performed a deep sub-clustering of the myeloid populations (Figure 6A). Annotations of populations were based on data from the primary study as well as recent publications.^32^^,^^33^^,^^34^ In addition to minor populations, previously characterized macrophage populations were identified, as shown in Figure 6A. While ELOVL5 was constitutively expressed in most macrophage populations, LPCAT3 was mostly enriched in foamy macrophages, including non-inflammatory macrophages (TREM2^hi^) (adjusted *p* value [padj]: 8.80E−25), and in the newly described population of pro-inflammatory foamy macrophages (TREM1^hi^/PLIN2^hi^) (padj: 7.76E−14) (Figure 6B).^33^ In parallel, we compared the expression profile between LPCAT3^+^ and LPCAT3^−^ plaque macrophages (Figure 6C). Importantly, the top genes enriched in LPCAT3^+^ macrophages are primarily involved in lipid metabolism, phagocytosis, and inflammatory response, indicating that these macrophages are well adapted to environments characterized by lipid accumulation or inflammatory stimuli, as seen in atherosclerosis (Figures 6C and 6D). The significant enrichment of LXR target genes (SMPDL3A, ABCA1, SCD, and LXRα) and the presence of scavenger receptors (CD36 and MARCO), along with genes involved in cholesterol handling (ABCA1, LIPA, NCEH1, and CYP27A1) and FA metabolism (SCD, FABP5, and Lp-PLA2), suggest that these macrophages play a key role in cholesterol and oxysterol regulation and in modulating lipid-mediated inflammation. This further supports the notion that LPCAT3 is an essential component of the LXR response in macrophages within the plaque, aiding their adaptation to the lipid-rich microenvironment.

Furthermore, we performed Mendelian randomization analysis to explore the potential causal relationship between the expression levels of LPCAT3 and ELOVL5 and the incidence of ischemic stroke in humans. *Cis*-expression quantitative trait loci (*cis-*eQTLs) associated with these genes in human blood were extracted from the eQTLGen database^35^ and used as exposures. We utilized genome-wide association study (GWAS) results from the Cohorts for Heart and Aging Research in Genomic Epidemiology (CHARGE) consortium^36^ as outcomes (Figure 6E). Our analysis revealed a significant negative causal effect of LPCAT3 expression in human whole blood on the risk of ischemic stroke, indicating that higher LPCAT3 expression is associated with a reduced likelihood of ischemic stroke (Figure 6F).

### Relationship between oxysterol, plasmalogen content, and plaque vulnerability in human carotid atheroma plaques

To further validate the clinical relevance of our observations, we took advantage of the Arachidonic Acid Metabolism in Carotid Stenosis Plaque in Diabetic Patients (MASCADI) study recently conducted by our team.^37^ The lipid composition of atheroma plaques from 187 patients who underwent carotid endarterectomy was determined.

**Figure 7:**
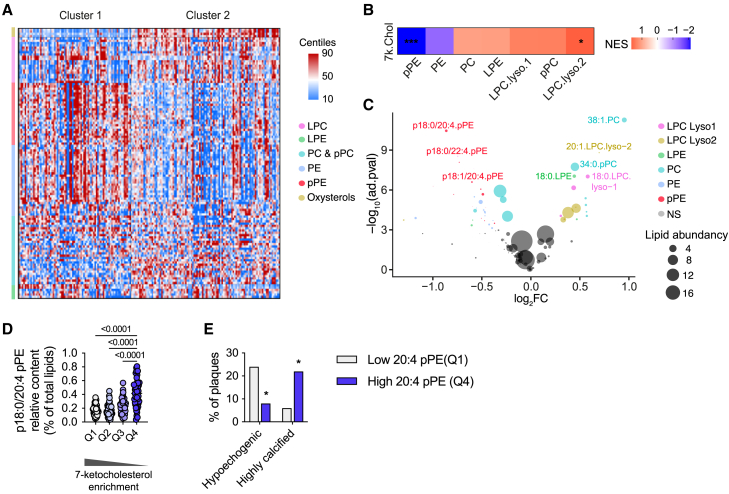
PUFA-enriched PLs are associated with favorable carotid plaque profile (A) Heatmap of major lipid molecules according to two carotid plaque clusters from the MASCADI cohort (cluster 1 *n* = 81; cluster 2 *n* = 106). Data are expressed as relative levels of lipid molecules. (B) Overall changes in lipid subclasses in plaques enriched or depleted with 7-ketocholesterol expressed with NES (normalized enrichment score). (C) Volcano plots of lipid molecules differentially enriched in carotid plaques above or below the median for 7-ketocholesterol content. (D) p18:0/20:4 pPE relative levels according to 7-ketocholesterol quartiles. (E) Echogenicity and calcification in plaques with either low (Q1) or high (Q4) 18:0/20:4 pPE content. Values are mean ± SD. Statistical analysis was performed with one-way ANOVA or Kruskal-Wallis, Student’s t test with adjust *p* values for volcano plot, or χ2 test for qualitative variables. For the heatmap, data normalization was performed by subtracting the median and dividing by the interquartile range (IQR). See also Tables S1 and S2.

Our previous data, which included oxysterols and lysophosphatidylcholines (LysoPCs), were implemented by the comprehensive analysis of all major classes of PLs, such as PE, PC, plasmalogens, sphingomyelins, and lysophosphatidylethanolamines (LysoPEs), using a liquid chromatography-tandem mass spectrometry (LC-MS/MS) approach. Total FAs were also determined by gas chromatography-mass spectrometry. The relative abundance of each lipid molecule within its own subclass was used for clustering analysis. For the clustering, a PCA was carried out followed by agglomerative hierarchical clustering. We choose to discriminate only two groups due the relatively small sample size (Table S1). Despite the clustering being based exclusively on the proportions of different lipid species, the two clusters corresponded to markedly different plaque profiles: hyperechoic and more calcified plaques typified cluster 1, whereas hypoechoic, and hence more vulnerable plaques, characterized cluster 2. This latter cluster also displayed a strong tendency toward a higher prevalence of symptomatic plaques, i.e., associated with recent strokes or transient ischemic attacks (*p* < 0.06) (Table S1). A broader analysis of the overall proportions of different lipid classes revealed that PEs and plasmalogens were significantly enriched in cluster 1, while lysophospholipids, PCs containing saturated FAs, and oxysterols emerged as the species most enriched in cluster 2 (Figure 7A; Table S2). Notably, out of all the lipid molecules, p18:0/20:4 and p18:0/22:4 pPE emerged among the most significantly enriched in cluster 1 (Table S2).

Concurrently, by employing a targeted approach, we investigated the lipid species particularly enriched or depleted in relation to 7-KC content of plaques (Figures 7B and 7C). Analogous to our observations with murine macrophages treated with 7-KC, 20:4 and 22:4 plasmalogens surfaced among the most depleted species in atheroma plaques with a high 7-KC content. Consequently, we discovered a strong negative association between 7-KC and p18:0/20:4 pPE content; the quartile with the lowest concentrations of 7-KC demonstrated twice as high proportions of p18:0/20:4 pPE (Figure 7D). Finally, we directly scrutinized the hypothesis of a correlation between p18:0/20:4 pPE content and the atheroma plaque morphological attributes. As shown in Figure 7E, plaques depleted in p18:0/20:4 pPE presented as more hypoechoic and less calcified, thus suggesting an enhanced plaque vulnerability (Figure 7E). Overall, these data corroborate the association of plasmalogens containing AA and oxysterols with human atheroma plaque vulnerability.

## Discussion

This study stemmed from the intriguing observation that, in *Lpcat3*^−/−^ macrophages, the reduction in C20 PUFAs, such as AA and EPA at the sn-2 position of PLs, was counteracted by a compensatory incorporation of C22 PUFAs. Here, we identified ELOVL5 as one elongase facilitating the conversion of C20 to C22 PUFA in *Lpcat3*-deficient cells by employing different experimental approaches with RAW 264.7 and primary macrophages. The role of ELOVL5 in PUFA elongation has primarily been characterized in the liver and more recently in lymphocytes.^38^^,^^39^^,^^40^^,^^41^ We show that *Elovl5* deletion significantly affects the PUFA profile of macrophages, accumulating FA species upstream of elongation steps.

Conceptually, we aimed to test the hypothesis that inactivating two targets facilitating PUFA incorporation into PLs could drastically reduce the PUFA content within cellular membranes (Figure S7). Results from the double *Lpcat3/Elovl5* deletion confirmed this hypothesis. *Lpcat3*^*Komac*^*/Elovl5*^−/−^ macrophages displayed a significant reduction in PLs containing C20 PUFAs without the compensatory increase in C22 FAs observed in *Lpcat3*^*Komac*^ macrophages along with a shift toward MUFA metabolism (Figure S7). It is well established that the type of FA chains composing biological membranes plays a pivotal role in determining their biophysical properties, including fluidity and the distribution of cholesterol and cholesterol-enriched domains. Saturated and MUFAs, due to their lower flexibility and greater affinity, are more prone to interact and form van der Waals bonds with cholesterol.^42^ The quantification of cholesterol-enriched domains revealed an increase in *Lpcat3*^*Komac*^*/Elovl5*^−/−^ macrophages compared to other genotypes, substantiating a direct association between PUFA content of membranes and cholesterol-enriched domain formation (Figure S7).

We focused on the macrophage response to oxysterols based on our observations in murine atheroma plaques. Increased necrotic cores and apoptotic cells were noted in *Ldlr*^−/−^ mice transplanted with hematopoietic cells from *Lpcat3*^*Komac*^*/Elovl5*^−/−^ mice. Oxysterols are abundant in atheroma plaques,^27^ and the apoptosis of macrophages induced by cytotoxic oxysterols is a well-documented process associated with DRMs.^29^ The incorporation of 7-KC within these micro-domains is crucial to oxysterol-induced apoptosis.^43^ Here, we show that *Lpcat3*^*Komac*^*/Elovl5*^−/−^ macrophages display heightened sensitivity to oxysterols. Moreover, treatment with 7-KC leads to membrane alterations and a depletion of lipid molecules generated by the LPCAT3-ELOVL5 axis, especially AA-containing plasmalogens. Plasmalogens, particularly those enriched in AA, are present in cholesterol-enriched domains and play a significant role in maintaining their integrity due to their unique structure.^44^^,^^45^ Therefore, the decrease in AA and ADA containing plasmalogens in *Lpcat3*^*Komac*^*/Elovl5*^−/−^ macrophages could render the cholesterol-enriched domains more vulnerable to 7-KC treatment, a molecule known for its DRM-destabilizing potential^46^ (Figure S7).

A second hypothesis centers on the antioxidant activity of plasmalogens through their plasmenyl linkage, which may limit peroxidation reactions and oxidative damage to other biological molecules,^47^ given that 7-KC is known to induce cellular oxidative stress.^43^ This mechanism would further elucidate the selective depletion of plasmalogens following treatment with 7-KC.

On a translational perspective, our analysis of human atheroma plaques reveals that PLs generated through the LPCAT3-ELOVL5 pathway, especially AA- or ADA-enriched plasmalogens, correlate with a more favorable plaque profile and show a negative association with oxysterol levels. This suggests their potential as valuable biomarkers in human studies. Additionally, Mendelian randomization analysis supports a protective role for LPCAT3, showing that higher LPCAT3 expression in human whole blood is causally linked to a reduced risk of ischemic stroke. Together, these findings highlight the therapeutic potential of targeting the LPCAT3-ELOVL5 axis for nutritional or pharmacological interventions in atherosclerosis. In this context, it is worth to note that AA-containing plasmalogens have been previously identified as markers of healthy obesity^44^ and that plasmalogen supplementation has been shown to attenuate atherosclerosis development in *apoE*-deficient mice.^48^

### Limitations of the study

Our study’s limitations include the need for further validation of human findings and examination of omega-3’s role in this pathway. In our experimental conditions, the concentrations of omega-3 FAs remain considerably lower than the level of omega-6. The potentially beneficial effects we observed in mice and in human atheroma plaques were mainly associated with PLs enriched in omega-6, particularly in AA. Although omega-6 FAs are often considered as pro-inflammatory, their role is undergoing reconsideration, with markers linked to the intake of omega-6 appearing beneficial in the context of cardiovascular diseases.^49^^,^^50^ Nevertheless, the specific impact of omega-3 FAs in our model will be important to investigate. Finally, the absence of sex stratification in the analysis may limit the generalizability of the findings and is acknowledged as a limitation.

## Resource availability

### Lead contact

Further information and requests for resources and reagents should be directed to and will be fulfilled by the lead contact David Masson (david.masson@u-bourgogne.fr).

### Materials availability

Materials generated in this study are available upon request to the lead contact.

### Data and code availability

All the data and details of analytical methods are available from the lead contact upon reasonable request. Accession codes for RNA-seq can be found in the key resources table. This paper does not report any original codes. Any additional information required to reanalyze the data reported in this work paper is available from the lead contact upon request.

## Acknowledgments

The authors greatly acknowledge the assistance of Amandine Bataille and Audrey Geissler from the histology platform Cellimap; Victoria Bergas and Hélène Choubley from the lipidomic analytic platform; Anabelle Sequeira-Le Grand, Nicolas Pernet, and Serge Monier from the cytometry platform; and Valérie Saint-Giorgio from the Centre de Zootechnie of the Université de Bourgogne for animal care. The authors would also like to thank Laure Avoscan, Elodie Noirot, and Chrystel Deulvot from the DimaCell platform for their help with confocal data acquisition, as well as for helpful discussions and technical advice. The authors would like to thank Dr. Tomasetto from Institut de Genetique et de Biologie Moléculaire et Cellulaire, Strasbourg, for the generous gift of the GFP-D4 probe. The authors acknowledge the CHARGE database for providing the GWAS summary statistics of the cardiovascular traits through dbGAP 10143.

This work was supported by the University Hospital of Dijon Bourgogne, by grants from the 10.13039/501100011773Conseil régional de Bourgogne-Franche-Comté, and by a French Government grant managed by the 10.13039/501100001665French National Research Agency under the program “appel à projet générique” with reference ANR-19-CE14-0020 (PUMAs project) and ANR-21-CE14-0084 (STATmiNADage) and the program “Investissements d’Avenir” with reference ANR-11-LABX-0021 (LipSTIC Labex). The funders had no role in the design of the study; in the collection, analyses, or interpretation of data; in the writing of the article, or in the decision to publish the results.

## Author contributions

Conceptualization, D.M. and A.J.. Methodology, D.M., C.T., T.P., and A.J. Data acquisition, interpretation, and statistical analysis: A.J., T.P., T.B., X.L., V.D., C.M., G.P., M.N., A.V., A.L., M.D., J.-P.P.d.B., N.L.G., F.H., L.P., V.A., E.L., E.S., and J.A. Writing – original draft, D.M., A.J., and T.P. Writing – review and editing and resources, C.T., L.L., N.V., and W.L.G. All the authors read and approved the final manuscript.

## Declaration of interests

The authors declare no competing interests.

## STAR★Methods

### Key resources table

**Table:** 

REAGENT or RESOURCE	SOURCE	IDENTIFIER
**Antibodies**		

Molecular Probes™ ProLong™ Diamond Antifade Mountant with DAPI	Thermo-Fisher	Cat# P36961
Mouse CD68 antibody | FA-11	Bio-Rad	Cat#MCA1957T; RRID:AB_2074849
Mouse FcR blocking reagent	Miltenyi	Cat#130-092-575; RRID:AB_2892833
Mouse CD115-PE	Miltenyi	Cat#130-112-639; RRID:AB_2654553
Mouse CD45 viogreen	Miltenyi	Cat#130-123-900; RRID:AB_2811572
Mouse Ly6C-APC	Miltenyi	Cat#130-111-917; RRID:AB_2652804

**Biological samples**		

Human carotid atheroma plaques	University Hospital of Dijon: department of Cardiovascular Surgery	MASCADI Study NCT03202823

**Chemicals, peptides, and recombinant proteins**		

Western diet	Harlan Teklad	Cat#TD88137
Enrofloxacin 0.25% Baytril ® 5%	Bayer	N/A
Human M-CSF	Miltenyi	Cat#130-096-492
18:0 PC	Sigma-Aldrich	Cat#850365C
Cholesterol	Sigma-Aldrich	Cat#C3045
C18:0(plasm)-20:4PE	Sigma-Aldrich	Cat#852804C
18:1 PS	Sigma-Aldrich	Cat#840035C
7-ketocholesterol	Sigma-Aldrich	Cat#700015P
DMSO	Santa Cruz Biotechnology	Cat#sc-359032
Cholesterol d7	Avanti Polar Lipids	Cat#700041
di17:0PC	Avanti Polar Lipids	Cat#850360
di17:0PE	Avanti Polar Lipids	Cat#830756
Isopropyl β-D-1-thiogalactopyranoside	Sigma -Aldrich	Cat#I5502
Protease Inhibitor	Roche	Cat#04693132001
HIS-Select® nickel affinity gel	Sigma-Aldrich	Cat#P6611
Lubrol 17A17	Serva	Cat#Serv.2807001

**Critical commercial assays**		

ApopTag Peroxidase *In Situ* Apoptosis Detection Kit	Sigma-Aldrich	Cat#S7100
CyQUANT™ LDH cytotoxicity assay	ThermoFisher	Cat#C20301
Lipofectamine 3000	ThermoFisher	Cat#15282465
Guide-it CRISPR/Cas9 System (Green)	Takara	Cat#632601
RNeasy Mini Kit	Qiagen	Cat#74106
High-Capacity cDNA Reverse Transcription Kit	Applied Biosystems	Cat#4368813
SYBRGreen®	Applied Biosystems	Cat#4367659
Cholesterol FS	DiaSys	Cat#1 1300 99 10 021
Triglycerides FS	DiaSys	Cat#1 5710 99 10 021

**Deposited data**		

Single-seq RNA-seq	https://doi.org/10.1161/ATVBAHA.123.320524; https://doi.org/10.1161/CIRCRESAHA.118.312804	GEO: GSE253904; GEO: GSE116240

**Experimental models: Cell lines**		

RAW 264.7	ATCC	RRID:CVCL_0493
Peripheral blood monocytes from human	Etablissement Français du Sang : healthy donors	N/A
Bone marrow derived macrophages from mice	Mus musculus C57Bl6N	Protocol n° 8381

**Experimental models: Organisms/strains**		

Mice Elovl5^Ko^ Lpcat3^Ko^ Lpcat3^Komac^ C57Bl6N	This Paper	N/A
Mice Ldlr^-/-^	University of Burgundy	N/A

**Software and algorithms**		

NDP.View	Hamamatsu	https://www.hamamatsu.com/eu/en/product/life-science-and-medical-systems/digital-slide-scanner/U12388-01.html
Flowjo v10	BD Biosciences	https://www.flowjo.com/solutions/flowjo/downloads
R studio	RStudio, Inc	https://www.r-studio.com/fr/
StepOnePlus	Aplied Biosystem	N/A
Prism 9	Graphpad	https://www.graphpad.com/features
Illustrator	Abobe	https://www.adobe.com/fr/products/illustrator.html
Excel	Microsoft	N/A
Image J	Schneider et al.	https://imagej.nih.gov/ij/
Zen	Zeiss	https://www.zeiss.com/microscopy/fr/produits/logiciel/zeiss-zen.html
Nanoscope Analysis	Bruker	https://www.brukersupport.com/BrukerDownloads/2
LasX	Leica	https://www.leica-microsystems.com/fr/produits/logiciel-du-microscope/p/leica-las-x-ls/downloads/
Nanosight Analysis	Malvern Panalytical	https://www.malvernpanalytical.com/en/support
Seurat V5	https://satijalab.org/seurat/	N/A
Metascape	https://metascape.org/	N/A
eQTLGen database	https://www.eqtlgen.org/	N/A
eQTL analysis	N/A	https://doi.org/10.1002/gepi.20576
CHARGE GWAS summary statistics	https://www.ncbi.nlm.nih.gov/projects/gap/cgi-bin/study.cgi?study_id=phs000930.v3.p1	dbGAP 10143
Mendelian randomization	https://doi.org/10.1002/gepi.22077	N/A
Linkage Disequilibrium	https://doi.org/10.1038/nature15393	Plink v1.90b6.21
MissMDA package	https://doi.org/10.32614/CRAN.package.missMDA	N/A
FactoMineR package	https://doi.org/10.32614/CRAN.package.FactoMineR	N/A
IDLE (Python 3.12) software	https://www.python.org/downloads/	N/A

**Others**		

GFP-D4 Probe	This Paper	N/A

### Experimental model and study participant details

#### Human study

The MASCADI protocol was reviewed and approved by the regional Ethics Committee (Comité de Protection des Personnes Est, Dijon, France CPP Est III, CHRU Nancy, N° 2017-A00022-51) and recorded on ClinicalTrials.gov (clinical registration number: NCT03202823). As described previously,^37^ patients enrolled in the present study were admitted to the Department of Cardiovascular Surgery at the University Hospital of Dijon (Burgundy, France) for the surgical treatment of an atheromatous internal carotid artery (ICA) stenosis whether symptomatic or not. A stenosis was considered symptomatic if stroke or transient ischemic attack (TIA) attributed to the ICA lesion occurred within the previous 6 months before intervention. According to the trial category (Interventional research that does not involve drugs or non-CE-marked medical devices and that involves only minimal risks and constraints for the patient) and the ethics committee, all patients received written information note on the study. Oral consent was obtained from the patient and an attestation of the patient’s oral consent was signed by the investigator physician and countersigned by the patient, according to the French law in this type of trial. For all patients, carotid endarterectomy (CEA) was performed within the carotid bulb, with en-bloc removal of the entire plaque, and resulting samples were frozen at -80°C prior to lipidomic analysis. A total of 187 patients (25.1% female; median age: [73.0] years) were included. Ancestry/Race/ethnicity were not recorded. Subjects were not randomized but retrospectively classified into two clusters based on unsupervised lipidomic profiling. Group assignment was therefore data-driven and not determined *a priori* by clinical characteristics. Sex was not significantly associated with cluster assignment. This absence of sex stratification in the analysis may limit the generalizability of the findings and is acknowledged as a limitation. Detailed participant characteristics, including sex distribution, are available in Table S1.

#### Mouse strain and breeding

*Lpcat3*^*-/-*^ and *Lpcat3*^*Komac*^ mice were described previously.^18^^,^^51^ *Elovl5*^*Ko*^ mice (Elovl5^Gt(CC0725)Wtsi)^) were obtained from the Mutant Mouse Regional Resource Center at UC Davis (Davis, CA) and were generated by inserting a genetrap cassette between the exon 3 and 4 of the *Elovl5* gene. *Lpcat3*^*flox/flox*^*/LysMCre*^*-*^*/Elovl5*^*+/-*^ mice were crossed with *Lpcat3*^*flox/flox*^*/LysMCre*^*+*^*/Elovl5*^*+/-*^ mice to obtain *Lpcat3*^*flox/flox*^*/LysMCre*^*-*^*/Elovl5*^*+/+*^ (WT), *Lpcat3*^*flox/flox*^*/LysMCre*^*+*^*/Elovl5*^*+/+*^ (*Lpcat3*^*Komac*^), *Lpcat3*^*flox/flox*^*/LysMCre*^*-*^*/Elovl5*^*-/-*^ (*Elovl5*^*-/-*^) and *Lpcat3*^*flox/flox*^*/LysMCre*^*+*^*/Elovl5*^*-/-*^ (*Lpcat3*^*Komac*^ *Elovl5*^*-/-*^) (Figure S2). We used male and female mice on a C57BL/6N background. All animal procedures were performed in accordance with institutional guidelines and approved by the University of Burgundy’s Ethics Committee on the Use of Laboratory Animals (protocol number #8381). At the age of 8-12 weeks old, mice were either maintained on a chow diet (A3, Safe), or a western diet for 12 weeks (TD88137, Harlan Teklad). The ARRIVE guidelines were followed throughout this study. The four groups of mice were compared, with individual animals serving as the experimental unit. The required sample size was determined using the Resource Equation Approach. All animals were included in the study. Each mouse was assigned an identification number prior to euthanasia, ensuring that the experimenter was blinded to the genotype. Euthanasia and organ collection were carried out consecutively under the same blinding conditions. This blinding was maintained during subsequent measurements and analyses. The link between the identification number and the genotype was only re-established after the procedures were completed. To assess potential sex-related differences, atherosclerosis outcomes were analyzed separately in male and female cohorts.

The origin of the mice, as well as all experimental procedures, are described in detail in the specific sections of the materials and methods («Mouse strain and breeding»; «Bone marrow transplantation»; «Atherosclerosis study»; «Bone marrow derived and fetal liver-derived macrophage preparation»).

#### Cell line

RAW 264.7 murine macrophage cell line was obtained from ATCC and transfected using Lipofectamine 3000 with plasmids expressing sgRNA targeting Elovl5. Cells were sorted based on GFP expression and clonally expanded. Cell lines were not authenticated but were validated by gene expression analysis of targeted genes. Cells were tested regularly for mycoplasma contamination.

### Method details

#### Bone marrow transplantation

Eight-week-old *Ldlr*^−/−^ mice were lethally irradiated with 1000 rads (11Gy) before transplantation. Recipient mice were injected with about 2 × 10^6^ bone marrow-derived monocytes through the tail vein. Recipient *Ldlr*^−/−^ mice were given acidified water (pH 4.5) containing enrofloxacin 0.25% for 3 weeks after transplantation.

#### Atherosclerosis study

After 4 weeks of recovery post-irradiation, the mice were fed a Western type diet (TD88137; Harlan Teklad) for 12 weeks. Mice were anesthetized using Isoflurane, blood was collected by intracardiac puncture and mice were killed by cervical dislocation. The hearts and proximal aortas were perfused with PBS, excised and fixed with paraformaldehyde. The tissues were serially sectioned in paraffin. The size of the aortic valve lesion of each animal was calculated as the mean lesion area of 3 sections by using NDP viewer software. Two investigators, blinded to the treatment received, independently analyzed the images. Three sections per mouse were used. TUNEL assay was performed on aortic valves using Apoptag peroxidase *in situ* apoptosis detection kit according to the manufacturer’s instructions (Sigma-Aldrich).

#### Bone marrow derived and fetal liver-derived macrophage preparation

Anesthetized mice were euthanized by cervical dislocation. Bone marrow in femur and tibia was flushed and 400.10^3^ bone marrow cells were implanted in 12 well plates. Fetal liver-derived macrophage preparation was performed as previously described.^18^ In both cases, cells were treated during 5-7 days with human M-CSF (130-096-492, Miltenyi) until full macrophage differentiation.

#### Liposome preparation

Liposomes were prepared as reported in the literature.^52^^,^^53^ For 18:0/18:0 PC we used a mix of 1.97mg/ml of 18:0 PC (850365C, Sigma), 0.4147mg/ml of CS (C3045, Sigma) and 0.86mg/ml of 18 :1 PS (840035C, Sigma) with a 7:3:3 ratio. For p18 :0/20 :4 pPE we used a mix of 0.98mg/ml of 18:0 PC (850365C, Sigma), 0.3534mg/ml of C18:0(plasm)-20:4 PE (852804C, Sigma), 0.4147mg/ml of CS (C3045, Sigma) and 0.86mg/ml of 18 :1 PS (840035C sigma) with a 3.5:3.5:3:3 ratio. The mixture was transferred to the rotary flask of a rotary evaporator and placed in a water bath set at a temperature 10°C above the phase transition temperature of each lipid. After 15 to 30 minutes of evaporation, the different mixtures were resuspended in 200 μL of PBS and returned to the rotary flask at the same temperature. Following a vortexing step, the mixtures were sealed under a nitrogen stream to prevent potential oxidation prior to cellular treatment. The size distribution was evaluated using a Nanosight analyzer. With diameters of approximately 100 nm, the liposomes were classified as intermediate, positioned between small unilamellar vesicles (SUVs) and large unilamellar vesicles (LUVs). Phosholipid profiling was performed on treated cells to confirm plasmalogen enrichment. More than 2-fold enrichment was constantly observed in all experiments (Figures S6A and S6B).

#### Cellular treatments

BMDMs were treated with two types of liposomes referred as 18:0 PC (Ctrl) or p18:0/20:4 pPE (plasmalogen enriched) for 24h at 37°C 5% CO2. The liposomes solutions were added at 1:200 in the final volume. Cells were treated with 7-ketocholesterol (700015P, Sigma) at a concentration range of 0 to 80μM for 18h at 37°C. For control conditions we used PBS or Ethanol. LDH assay was performed to assess 7-ketocholesterol toxicity according to manufacturer instructions (C20301, Thermofisher).

#### Plasma lipid analysis

The plasma lipid parameters were determined on a Victor2 1420 Multilabel Counter (PerkinElmer Life Science, Boston, MA). Total cholesterol and TG concentrations were measured by colorimetric enzymatic methods as previously described.^18^

#### Monocyte phenotyping

100μL of blood were collected by retro-orbital puncture and hemolyzed in 5mL of red blood cell lysis buffer for 5 min at room temperature. Cells were then centrifugated (300g, 5min, 4°C) and washed with PBS without calcium and magnesium containing 2mM EDTA (PBS w/o Ca^2+^-Mg^2+^). Cells were then resuspended in PBS w/o Ca^2+^-Mg^2+^ containing FcR blocking reagent according to manufacturer’s instructions (Miltenyi). Cells were immunostained with CD45-Viogreen, CD115-PE, Ly6C-APC antibodies according to manufacturer’s instructions (Milenyi Biotec). Cells were then wash and resuspended in PBS w/o Ca^2+^-Mg^2+^ and kept at 4°C until flow cytometry. Flow cytometry was performed on LSR Fortessa (BD biosciences). No FMO controls were used. Then, data were analyzed on Flow Jo v10. Total blood cell counts were performed on an automated analyzer (Scil Vet Abc Plus).

#### Materials for lipidomic analysis

Phosphatidylcholines, phosphatidylethanolamines and plasmenylethanolamines, cholesterol (cholesterol, di17:0-PC, di17:0-PE) were obtained from Avanti Polar Lipids (Coger SAS, Paris, France). LCMS/MS quality grade solvents were purchased from Fischer Scientific (Illkirch, France). Other chemicals of the highest grade available were purchased from Sigma Aldrich (Saint-Quentin Fallavier, France). Macrophages pellets (10.10^6^ cells) were spiked with a lipid-standard mix containing 0,1 μg of di17:0-PC, 0,05 μg of di17:0-PE used as internal standards. Total lipids were extracted according to the method of Folch et al..^54^

#### Phospholipids quantitation by LCMS/MS

Phosphatidylcholines, phosphatidylethanolamines and plasmenylethanolamines were analyzed by LC-MS/MS using the same chromatographic conditions as previously described.^55^ Acquisition was performed on an Agilent 6460 QqQ mass spectrometer in positive selected reaction monitoring ion mode (source temperature 325°C, nebulizer gas flow rate 10 L/min, sheath gas flow 11 L/min, temperature 300°C, capillary 3500 V, nozzle 1000 V). Fragmentor was set up at 136 V and 160 V for ethanolamines and phosphatidylcholines respectively. Collision energy was set up at 12 V and 20 V for ethanolamines and phosphatidylcholines respectively. Each glycerophospholipid was semi-quantitated by calculating their response ratio with regard to their respective internal standard.

#### Total cholesterol quantitation by GCMS

Once cholesteryl esters and phospholipids analysis by LC-MS/MS was achieved, samples were evaporated under vacuum. Lipids were saponified for 45mn at 56°C with 60mL of potassium hydroxide 10mol/L and 1.2 mL of ethanol. The resulting unesterified sterols were extracted with 5mL of hexane and 1mL of water. After evaporation of the organic phase, sterols were derivatized with 100mL of a mixture of bis(trimethylsilyl)trifluoroacetamide/ trimethylchlorosilane 4/1 v/v for one hour at 80°C. After evaporation of the silylating reagent 100mL of hexane were added. Derivatized sterols were analyzed by GCMS in a 6890 gas chromatograph coupled with a 7673 Mass Detector (Agilent Technologies). Separation was achieved on a HP-5MS 30m x 250μm column (Agilent Technologies) using helium as carrier gas and the following GC conditions: injection at 250 °C using the split mode, oven temperature program: initial temperature 140°C up to 280°C at a rate of 15°C/min, up to 300°C at a rate of 2°C/min. The MSD was set up as follow: EI at 70 eV mode, source temperature at 230°C. Data were acquired in SIM mode using 368.3 quantitation ions (m/z) for cholesterol. A calibration curve was obtained with cholesterol standard using the same method used for samples. Free cholesterol was calculated as the difference between total and esterified cholesterol.

#### Plasma membrane staining

For flow cytometry, 4.10^5^ cells were incubated in a solution of GFP-D4 (1: 200 in 1x PBS) for 30min at 37°C. After a wash, cells were then fixed with 0.1% paraformaldehyde in PBS, centrifuged, and finally resuspended in PBS before FACS analysis (LSR Fortessa, BD biosciences). For microscope acquisitions, 2.5.10^5^ cells were grown on glass coverslips, treated and incubated as described above. After a rapid wash, cells were mounted in ProLong Diamond (P36961, Invitrogen).

#### Isolation of DRMs

20.10^6^ cells were washed with PBS, then lysed on ice for 30min in 2ml of MBS buffer (Sigma) containing 1% of Lubrol WX (Serva, Paris, France). Lysates were homogenized with mechanical stress with a 26Gx syringe. Lysates were then mixed with sucrose solution in order to obtain a final concentration of 45% sucrose. The sucrose density gradient of 4ml of sucrose at 45%, 5ml of sucrose at 35% and 3ml of sucrose at 5% was prepared in MBS buffer. The gradient was centrifuged at 39,000g, 20h, 4°C. Twelve fractions of 1ml were collected from the top to the bottom, vortexed and stored at -80°C before lipid and protein analysis.

#### CrispR-Cas9 raw 264.7 cell generation

Raw 264.7 were transfected using Lipofectamin 3000 (Invitrogen) with pGuide-it Vector previously annealed with Elovl5 sgRNA (Clontech laboratories). Positive cells were sorted by a GFP selection using a FACS ARIA III (BD biosciences). GFP positive cells were isolated in 96 wells in order to generate a cell line from each clone.

#### Real-Time PCR

Cell culture lysates were directly stored at -80°C. Total RNA was isolated using RNeasy Mini Kit (74106, Qiagen) and quantified by spectrophotometer (Nanodrop 1000, Thermo Scientific). One hundred to 1000ng RNA was reverse transcribed using High-Capacity cDNA Reverse Transcription Kit (Multiscribe® reverse transcriptase, 4368813, Applied Biosystems) and quantitative PCR were performed using StepOnePlus (Real-Time PCR System, Applied Biosystems) and SYBRGreen® (4367659, Applied Biosystems) technologies. The mRNA levels were normalized with housekeeping gene *Gapdh* and expressed as relative expression using the 2^-ΔΔCt^ method.

#### Confocal microscopy

A Leica TCS SP8 MP Multiphoton Microscope was used to perform confocal microscopy. The images were acquired with bidirectional scan direction of 600Hz at x63 (HC PL APO CS2 63x/1.40 OIL) and a pinhole of 95.5 μm. Two lasers were used: 405nm for DAPI and 488nm for GFP-D4. A x5 numeric zoom and the Z stack option were used to provide 3D images (1024x1024) then analyzed with the LasXTM software (Leica). For 3D images, the LasXTM software automatically applied frame averaging, combining more than 20 images to reduce random noise and enhance image quality. A total of four cells per mice were obtained and quantified with Image JTM software (NIH) following the maximum projection of images acquired in Z-stack mode. To limit the autofluorescence of the GFP-D4 probe, the power of the 488nm laser was set at 0.6005% intensity.

#### Atomic force microscopy

An atomic force microscope (AFM) multimode 8 equipped with a 15 μm range scanner and a nanoscope V controller (Bruker AXS, Santa Barbara, USA) was used for AFM measurements. Images were acquired using the PeakForce tapping mode in MES [20 mM] – NaCl [140mM] pH5.8 solution with a silicon cantilever (ScanAsyst-Air-HR probe, k= 0.2-0.3 N/m, 2-nm nominal tip radius, Bruker probes, Camarillo, USA) and a set-point value of 0.050 V in non-automated ScanAsyst mode. A drop of 0.5 μl of purified DRMs (0.1mg/ml) was deposited on freshly cleaved mica 1.5mm disk (RIBM, Japan) and allow to adsorb for 10 minutes at room temperature. The samples were rinsed two times and were imaged with 150 μl of MES buffer solution. Series of more than 30 raw AFM images per sample was obtained at a scan rate of 1Hz with a physical size of 0.64 and 4 μm^2^ and 1024 × 1024 pixels. The images were pretreated with first-order polynomial background and horizontal stroke corrections performed using NanoScope Analysis software (Bruker).

#### Single cell RNA-seq analysis

Single-cell RNA-seq data generated by CellRanger were obtained from the GSE253904 and GSE116240 dataset.^26^^,^^32^ Data were processed and analyzed using Seurat v5 following the recommended integration pipeline. The IntegrateLayers function was applied using the RPCA method. Differential gene expression analysis between clusters was conducted using the FindMarkers function with default settings. Genes with adjusted p-values (padj < 0.05) were considered significantly enriched. Significantly enriched gene ontology terms were calculated by using differentially expressed genes by using Metascape (www.metascape.org).^56^

#### Mendelian randomization (MR) analysis

We extracted *cis*-expression quantitative trait loci (*cis-*eQTLs) of *LPCAT3* and *ELOVL5* in whole blood from the eQTLGen^35^ database (window size 1Mb, MAF ≥1%) and then restricted them to those also present in the Genome Wide Association Study (GWAS) result from the Cohorts for Heart and Aging Research in Genomic Epidemiology (CHARGE),^36^ which is used as exposures. The effect sizes of these *cis*-eQTLs were estimated based on the Z-score, the reported allele frequency and the sample size using the method described in Vukcevic et al.^57^

We are authorized to access the CHARGE GWAS summary statistics through dbGAP project 10143. The GWAS summary statistics for the incidence ischemic stroke in subjects with European ancestry in this database were used as outcomes. MR was performed using the PCA-based method proposed by Burgess et al.^58^ The Linkage Disequilibrium (LD) structure for the PCA was estimated using Plink v1.90b6.21^59^ on the 1000 Genomes reference panel. Principal Components (PCs) were selected to explain at least 99% of the variation, resulting in 6 PCs for *LPCAT3* and 4 PCs for *ELOVL5*.

### Quantification and statistical analysis

#### Statistical analysis

The number of replicates (n) per experiment was determined based on standard practices in the field and previous publications from our group assessing similar endpoints. For most of the analyses, normality was assessed by Shapiro-wilk test. Significance of the data was determined using one-way ANOVA or Kruskal-Wallis test for more than two groups, and by student t or Mann-Whitney test for comparison of two groups. Correction for multiple comparison was performed by the Two-stage linear step-up procedure of Benjamini, Krieger and Yekutieli. Data are presented as mean ± SD or median +/- 95% CI. Statistical analyses were performed using GraphPad Prism 9. A value of p < 0.05 was considered statistically significant.

Principal Component Analysis (PCA) was performed using singular value decomposition. Analyses were conducted in Python, employing the pandas library for data manipulation, scikit-learn for normalization and PCA, and matplotlib for visualizations.

For the MASCADI study, missing data were imputed using a PCA model, specifically the impute PCA function from the MissMDA package. A PCA was then performed on the selected variables using the PCA function from the FactoMineR package. Following this, an agglomerative hierarchical clustering (HCPC function, FactoMineR package) was applied to the PCA results, leading to a two-cluster solution. The two clusters were compared using the Shapiro-Wilk normality test, followed by appropriate statistical tests such as t-tests, Mann-Whitney tests, Chi-square tests, or Fisher's exact tests depending on the distribution of the variables.

### Additional resources

Clinical Registry Number and link : https://clinicaltrials.gov/search?term=NCT03202823.

## Supplemental information

Document S1. Figures S1–S7 and Tables S1–S3

Document S2. Article plus supplemental information

## References

[1] Chawla A., Nguyen K.D., Goh Y.P.S. (2011). Macrophage-mediated inflammation in metabolic disease. Nat. Rev. Immunol..

[2] Kazankov K., Jørgensen S.M.D., Thomsen K.L., Møller H.J., Vilstrup H., George J., Schuppan D., Grønbæk H. (2019). The role of macrophages in nonalcoholic fatty liver disease and nonalcoholic steatohepatitis. Nat. Rev. Gastroenterol. Hepatol..

[3] Koelwyn G.J., Corr E.M., Erbay E., Moore K.J. (2018). Regulation of macrophage immunometabolism in atherosclerosis. Nat. Immunol..

[4] Ménégaut L., Jalil A., Thomas C., Masson D. (2019). Macrophage fatty acid metabolism and atherosclerosis: The rise of PUFAs. Atherosclerosis.

[5] Kanter J.E., Kramer F., Barnhart S., Averill M.M., Vivekanandan-Giri A., Vickery T., Li L.O., Becker L., Yuan W., Chait A. (2012). Diabetes promotes an inflammatory macrophage phenotype and atherosclerosis through acyl-CoA synthetase 1. Proc. Natl. Acad. Sci. USA.

[6] Fredman G., Hellmann J., Proto J.D., Kuriakose G., Colas R.A., Dorweiler B., Connolly E.S., Solomon R., Jones D.M., Heyer E.J. (2016). An imbalance between specialized pro-resolving lipid mediators and pro-inflammatory leukotrienes promotes instability of atherosclerotic plaques. Nat. Commun..

[7] Shaikh S.R., Kinnun J.J., Leng X., Williams J.A., Wassall S.R. (2015). How polyunsaturated fatty acids modify molecular organization in membranes: Insight from NMR studies of model systems. Biochim. Biophys. Acta.

[8] Hashimoto M., Hossain S., Yamasaki H., Yazawa K., Masumura S. (1999). Effects of eicosapentaenoic acid and docosahexaenoic acid on plasma membrane fluidity of aortic endothelial cells. Lipids.

[9] Pinot M., Vanni S., Pagnotta S., Lacas-Gervais S., Payet L.-A., Ferreira T., Gautier R., Goud B., Antonny B., Barelli H. (2014). Lipid cell biology. Polyunsaturated phospholipids facilitate membrane deformation and fission by endocytic proteins. Science.

[10] Cawood A.L., Ding R., Napper F.L., Young R.H., Williams J.A., Ward M.J.A., Gudmundsen O., Vige R., Payne S.P.K., Ye S. (2010). Eicosapentaenoic acid (EPA) from highly concentrated n−3 fatty acid ethyl esters is incorporated into advanced atherosclerotic plaques and higher plaque EPA is associated with decreased plaque inflammation and increased stability. Atherosclerosis.

[11] Endres S., Ghorbani R., Kelley V.E., Georgilis K., Lonnemann G., van der Meer J.W., Cannon J.G., Rogers T.S., Klempner M.S., Weber P.C. (1989). The Effect of Dietary Supplementation with n—3 Polyunsaturated Fatty Acids on the Synthesis of Interleukin-1 and Tumor Necrosis Factor by Mononuclear Cells. N. Engl. J. Med..

[12] Jalil A., Bourgeois T., Ménégaut L., Lagrost L., Thomas C., Masson D. (2019). Revisiting the Role of LXRs in PUFA Metabolism and Phospholipid Homeostasis. Int. J. Mol. Sci..

[13] Köberlin M.S., Snijder B., Heinz L.X., Baumann C.L., Fauster A., Vladimer G.I., Gavin A.-C., Superti-Furga G. (2015). A Conserved Circular Network of Coregulated Lipids Modulates Innate Immune Responses. Cell.

[14] Wei X., Song H., Yin L., Rizzo M.G., Sidhu R., Covey D.F., Ory D.S., Semenkovich C.F. (2016). Fatty acid synthesis configures the plasma membrane for inflammation in diabetes. Nature.

[15] Hishikawa D., Shindou H., Kobayashi S., Nakanishi H., Taguchi R., Shimizu T. (2008). Discovery of a lysophospholipid acyltransferase family essential for membrane asymmetry and diversity. Proc. Natl. Acad. Sci. USA.

[16] Harayama T., Eto M., Shindou H., Kita Y., Otsubo E., Hishikawa D., Ishii S., Sakimura K., Mishina M., Shimizu T. (2014). Lysophospholipid acyltransferases mediate phosphatidylcholine diversification to achieve the physical properties required in vivo. Cell Metab..

[17] Shindou H., Hishikawa D., Harayama T., Eto M., Shimizu T. (2013). Generation of membrane diversity by lysophospholipid acyltransferases. J. Biochem..

[18] Thomas C., Jalil A., Magnani C., Ishibashi M., Queré R., Bourgeois T., Bergas V., Ménégaut L., Patoli D., Le Guern N. (2018). LPCAT3 deficiency in hematopoietic cells alters cholesterol and phospholipid homeostasis and promotes atherosclerosis. Atherosclerosis.

[19] Rong X., Albert C.J., Hong C., Duerr M.A., Chamberlain B.T., Tarling E.J., Ito A., Gao J., Wang B., Edwards P.A. (2013). LXRs regulate ER stress and inflammation through dynamic modulation of membrane phospholipid composition. Cell Metab..

[20] Ishibashi M., Varin A., Filomenko R., Lopez T., Athias A., Gambert P., Blache D., Thomas C., Gautier T., Lagrost L., Masson D. (2013). Liver x receptor regulates arachidonic acid distribution and eicosanoid release in human macrophages: a key role for lysophosphatidylcholine acyltransferase 3. Arterioscler. Thromb. Vasc. Biol..

[21] Jiang H., Li Z., Huan C., Jiang X.-C. (2018). Macrophage Lysophosphatidylcholine Acyltransferase 3 Deficiency-Mediated Inflammation Is Not Sufficient to Induce Atherosclerosis in a Mouse Model. Front. Cardiovasc. Med..

[22] Jakobsson A., Westerberg R., Jacobsson A. (2006). Fatty acid elongases in mammals: their regulation and roles in metabolism. Prog. Lipid Res..

[23] Brzustowicz M.R., Cherezov V., Caffrey M., Stillwell W., Wassall S.R. (2002). Molecular Organization of Cholesterol in Polyunsaturated Membranes: Microdomain Formation. Biophys. J..

[24] Shaw A.S. (2006). Lipid rafts: now you see them, now you don’t. Nat. Immunol..

[25] Ohno-Iwashita Y., Shimada Y., Waheed A.A., Hayashi M., Inomata M., Nakamura M., Maruya M., Iwashita S. (2004). Perfringolysin O, a cholesterol-binding cytolysin, as a probe for lipid rafts. Anaerobe.

[26] Kim K., Shim D., Lee J.S., Zaitsev K., Williams J.W., Kim K.-W., Jang M.-Y., Seok Jang H., Yun T.J., Lee S.H. (2018). Transcriptome Analysis Reveals Nonfoamy Rather Than Foamy Plaque Macrophages Are Proinflammatory in Atherosclerotic Murine Models. Circ. Res..

[27] Brown A.J., Jessup W. (1999). Oxysterols and atherosclerosis. Atherosclerosis.

[28] Royer M.-C., Lemaire-Ewing S., Desrumaux C., Monier S., Pais de Barros J.P., Athias A., Néel D., Lagrost L. (2009). 7-Ketocholesterol Incorporation into Sphingolipid/Cholesterol-enriched (Lipid Raft) Domains Is Impaired by Vitamin E: A SPECIFIC ROLE FOR α-TOCOPHEROL WITH CONSEQUENCES ON CELL DEATH. J. Biol. Chem..

[29] Berthier A., Lemaire-Ewing S., Prunet C., Monier S., Athias A., Bessède G., Pais de Barros J.-P., Laubriet A., Gambert P., Lizard G., Néel D. (2004). Involvement of a calcium-dependent dephosphorylation of BAD associated with the localization of Trpc-1 within lipid rafts in 7-ketocholesterol-induced THP-1 cell apoptosis. Cell Death Differ..

[30] Varin A., Thomas C., Ishibashi M., Ménégaut L., Gautier T., Trousson A., Bergas V., de Barros J.P.P., Narce M., Lobaccaro J.M.A. (2015). Liver X receptor activation promotes polyunsaturated fatty acid synthesis in macrophages: relevance in the context of atherosclerosis. Arterioscler. Thromb. Vasc. Biol..

[31] Terasaka N., Wang N., Yvan-Charvet L., Tall A.R. (2007). High-density lipoprotein protects macrophages from oxidized low-density lipoprotein-induced apoptosis by promoting efflux of 7-ketocholesterol via ABCG1. Proc. Natl. Acad. Sci. USA.

[32] Bashore A.C., Yan H., Xue C., Zhu L.Y., Kim E., Mawson T., Coronel J., Chung A., Sachs N., Ho S. (2024). High-Dimensional Single-Cell Multimodal Landscape of Human Carotid Atherosclerosis. Arterioscler. Thromb. Vasc. Biol..

[33] Dib L., Koneva L.A., Edsfeldt A., Zurke Y.-X., Sun J., Nitulescu M., Attar M., Lutgens E., Schmidt S., Lindholm M.W. (2023). Lipid-associated macrophages transition to an inflammatory state in human atherosclerosis increasing the risk of cerebrovascular complications. Nat. Cardiovasc. Res..

[34] Slysz J., Sinha A., DeBerge M., Singh S., Avgousti H., Lee I., Glinton K., Nagasaka R., Dalal P., Alexandria S. (2023). Single-cell profiling reveals inflammatory polarization of human carotid versus femoral plaque leukocytes. JCI Insight.

[35] Võsa U., Claringbould A., Westra H.-J., Bonder M.J., Deelen P., Zeng B., Kirsten H., Saha A., Kreuzhuber R., Yazar S. (2021). Large-scale cis- and trans-eQTL analyses identify thousands of genetic loci and polygenic scores that regulate blood gene expression. Nat. Genet..

[36] Psaty B.M., O’Donnell C.J., Gudnason V., Lunetta K.L., Folsom A.R., Rotter J.I., Uitterlinden A.G., Harris T.B., Witteman J.C.M., Boerwinkle E., CHARGE Consortium (2009). Cohorts for Heart and Aging Research in Genomic Epidemiology (CHARGE) Consortium: Design of prospective meta-analyses of genome-wide association studies from five cohorts. Circ. Cardiovasc. Genet..

[37] Ménégaut L., Laubriet A., Crespy V., Leleu D., Pilot T., Van Dongen K., de Barros J.-P.P., Gautier T., Petit J.-M., Thomas C. (2023). Inflammation and oxidative stress markers in type 2 diabetes patients with Advanced Carotid atherosclerosis. Cardiovasc. Diabetol..

[38] Moon Y.-A., Hammer R.E., Horton J.D. (2009). Deletion of ELOVL5 leads to fatty liver through activation of SREBP-1c in mice. J. Lipid Res..

[39] Robichaud P.-P., Munganyiki J.E., Boilard E., Surette M.E. (2018). Polyunsaturated fatty acid elongation and desaturation in activated human T-cells: ELOVL5 is the key elongase. J. Lipid Res..

[40] Wang Y., Botolin D., Xu J., Christian B., Mitchell E., Jayaprakasam B., Nair M.G., Peters J.M., Busik J.V., Olson L.K., Jump D.B. (2006). Regulation of hepatic fatty acid elongase and desaturase expression in diabetes and obesity. J. Lipid Res..

[41] Wang Y., Torres-Gonzalez M., Tripathy S., Botolin D., Christian B., Jump D.B. (2008). Elevated hepatic fatty acid elongase-5 activity affects multiple pathways controlling hepatic lipid and carbohydrate composition. J. Lipid Res..

[42] Levental I., Levental K.R., Heberle F.A. (2020). Lipid Rafts: Controversies Resolved, Mysteries Remain. Trends Cell Biol..

[43] Nury T., Yammine A., Ghzaiel I., Sassi K., Zarrouk A., Brahmi F., Samadi M., Rup-Jacques S., Vervandier-Fasseur D., Pais de Barros J.P. (2021). Attenuation of 7-ketocholesterol- and 7β-hydroxycholesterol-induced oxiapoptophagy by nutrients, synthetic molecules and oils: Potential for the prevention of age-related diseases. Ageing Res. Rev..

[44] Pietiläinen K.H., Róg T., Seppänen-Laakso T., Virtue S., Gopalacharyulu P., Tang J., Rodriguez-Cuenca S., Maciejewski A., Naukkarinen J., Ruskeepää A.-L. (2011). Association of lipidome remodeling in the adipocyte membrane with acquired obesity in humans. PLoS Biol..

[45] Pike L.J., Han X., Chung K.-N., Gross R.W. (2002). Lipid Rafts Are Enriched in Arachidonic Acid and Plasmenylethanolamine and Their Composition Is Independent of Caveolin-1 Expression: A Quantitative Electrospray Ionization/Mass Spectrometric Analysis. Biochemistry.

[46] Wnętrzak A., Chachaj-Brekiesz A., Stępniak A., Kobierski J., Dynarowicz-Latka P. (2022). Different effects of oxysterols on a model lipid raft - Langmuir monolayer study complemented with theoretical calculations. Chem. Phys. Lipids.

[47] Engelmann B. (2004). Plasmalogens: targets for oxidants and major lipophilic antioxidants. Biochem. Soc. Trans..

[48] Rasmiena A.A., Barlow C.K., Stefanovic N., Huynh K., Tan R., Sharma A., Tull D., de Haan J.B., Meikle P.J. (2015). Plasmalogen modulation attenuates atherosclerosis in ApoE- and ApoE/GPx1-deficient mice. Atherosclerosis.

[49] Harris W.S., Mozaffarian D., Rimm E., Kris-Etherton P., Rudel L.L., Appel L.J., Engler M.M., Engler M.B., Sacks F. (2009). Omega-6 Fatty Acids and Risk for Cardiovascular Disease. Circulation.

[50] Marklund M., Wu J.H.Y., Imamura F., Del Gobbo L.C., Fretts A., de Goede J., Shi P., Tintle N., Wennberg M., Aslibekyan S. (2019). Biomarkers of Dietary Omega-6 Fatty Acids and Incident Cardiovascular Disease and Mortality. Circulation.

[51] Bourgeois T., Jalil A., Thomas C., Magnani C., Le Guern N., Gautier T., Pais de Barros J.-P., Bergas V., Choubley H., Mazzeo L. (2021). Deletion of lysophosphatidylcholine acyltransferase 3 in myeloid cells worsens hepatic steatosis after a high-fat diet. J. Lipid Res..

[52] Lombardo D., Kiselev M.A. (2022). Methods of Liposomes Preparation: Formation and Control Factors of Versatile Nanocarriers for Biomedical and Nanomedicine Application. Pharmaceutics.

[53] Schwendener R.A., Schott H. (2010). Liposome formulations of hydrophobic drugs. Methods Mol. Biol..

[54] Folch J., Lees M., Sloane Stanley G.H. (1957). A simple method for the isolation and purification of total lipides from animal tissues. J. Biol. Chem..

[55] Vial G., Chauvin M.-A., Bendridi N., Durand A., Meugnier E., Madec A.-M., Bernoud-Hubac N., Pais de Barros J.-P., Fontaine É., Acquaviva C. (2015). Imeglimin normalizes glucose tolerance and insulin sensitivity and improves mitochondrial function in liver of a high-fat, high-sucrose diet mice model. Diabetes.

[56] Zhou Y., Zhou B., Pache L., Chang M., Khodabakhshi A.H., Tanaseichuk O., Benner C., Chanda S.K. (2019). Metascape provides a biologist-oriented resource for the analysis of systems-level datasets. Nat. Commun..

[57] Vukcevic D., Hechter E., Spencer C., Donnelly P. (2011). Disease model distortion in association studies. Genet. Epidemiol..

[58] Burgess S., Zuber V., Valdes-Marquez E., Sun B.B., Hopewell J.C. (2017). Mendelian randomization with fine-mapped genetic data: Choosing from large numbers of correlated instrumental variables. Genet. Epidemiol..

[59] Auton A., Abecasis G.R., Altshuler D.M., Durbin R.M., Abecasis G.R., Bentley D.R., Chakravarti A., Clark A.G., Donnelly P., Eichler E.E. (2015). A global reference for human genetic variation. Nature.

