# Supplementary material for: Plasmalogen remodeling modulates macrophage response to cytotoxic oxysterols and atherosclerotic plaque vulnerability
Source: Cell Rep Med. 2025 May 8;6(5):102131. doi: 10.1016/j.xcrm.2025.102131 (PMC12147908; doi:10.1016/j.xcrm.2025.102131)
Supplement: Document S1. Figures S1–S7 and Tables S1–S3 [file mmc1.pdf]

Cell Reports Medicine, Volume *6*

Supplemental information

Plasmalogen remodeling modulates macrophage response to cytotoxic oxysterols and atherosclerotic plaque vulnerability

Antoine Jalil, Thomas Pilot, Thibaut Bourgeois, Aline Laubriet, Xiaoxu Li, Marc Diedisheim, Valérie Deckert, Charlène Magnani, Naig Le Guern, Jean-Paul Pais de Barros, Maxime Nguyen, Gaëtan Pallot, Adrien Vouilloz, Lil Proukhnitzky, François Hermetet, Virginie Aires, Eric Lesniewska, Laurent Lagrost, Johan Auwerx, Wilfried Le Goff, Nicolas Venteclef, Eric Steinmetz, Charles Thomas, and David Masson

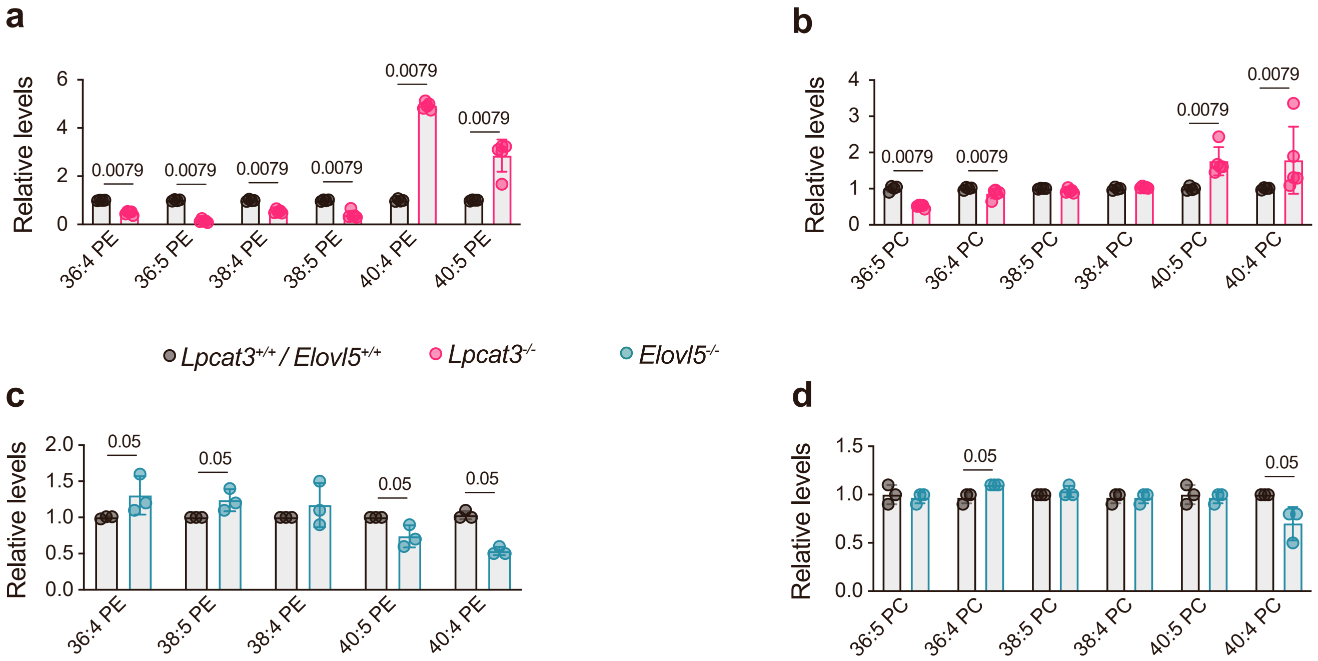

Figure S1. Distinct roles of Elovl5 and Lpcat3 in PL remodeling in murine macrophages. *Related to Figure 1.* (A.B). Amounts of PCs and PEs containing C20:4 n-6, C22:4 n-6, C20:5 n-3 and C22:5 n-3 in *Lpcat3*$^{+/+}$ and *Lpcat3*$^{-/-}$ macrophages. Data are expressed as percentage of total PEs or PCs and are normalized as 1 in *Lpcat3*$^{+/+}$ group (n=4). (C.D). Amounts of PCs and PEs containing C20:4 n-6, C22:4 n-6, C20:5 n-3 and C22:5 n-3 in *Elovl5*$^{+/+}$ and *Elovl5*$^{-/-}$ macrophages. Data are expressed as percentage of total PEs or PCs and are normalized as 1 in *Elovl5*$^{+/+}$ group (n=3). Values are mean ± SD. Statistical analysis performed with unpaired t-test or Mann-Whitney.

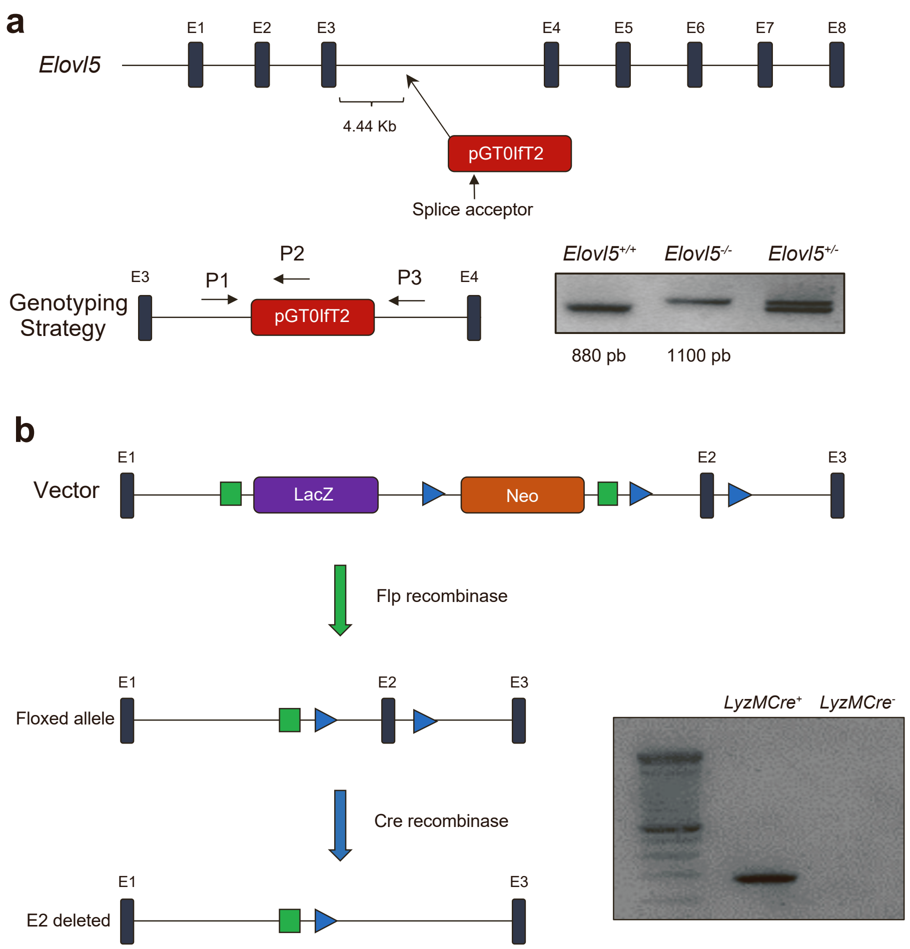

Figure S2. Genetic construction of Elovl5$^{-/-}$ & Lpcat3^{Komac}. *Related to Figure 2.* (A). *Elovl5* targeting vector. A gene-trap pGT0IfT2 is located upstream of exon 4 of *Elovl5* gene. (B). *Lpcat3* targeting vector. A gene-trap LacZ-cassette is located downstream of exon 3 of *Lpcat3* gene. Validation of genotype by PCR.

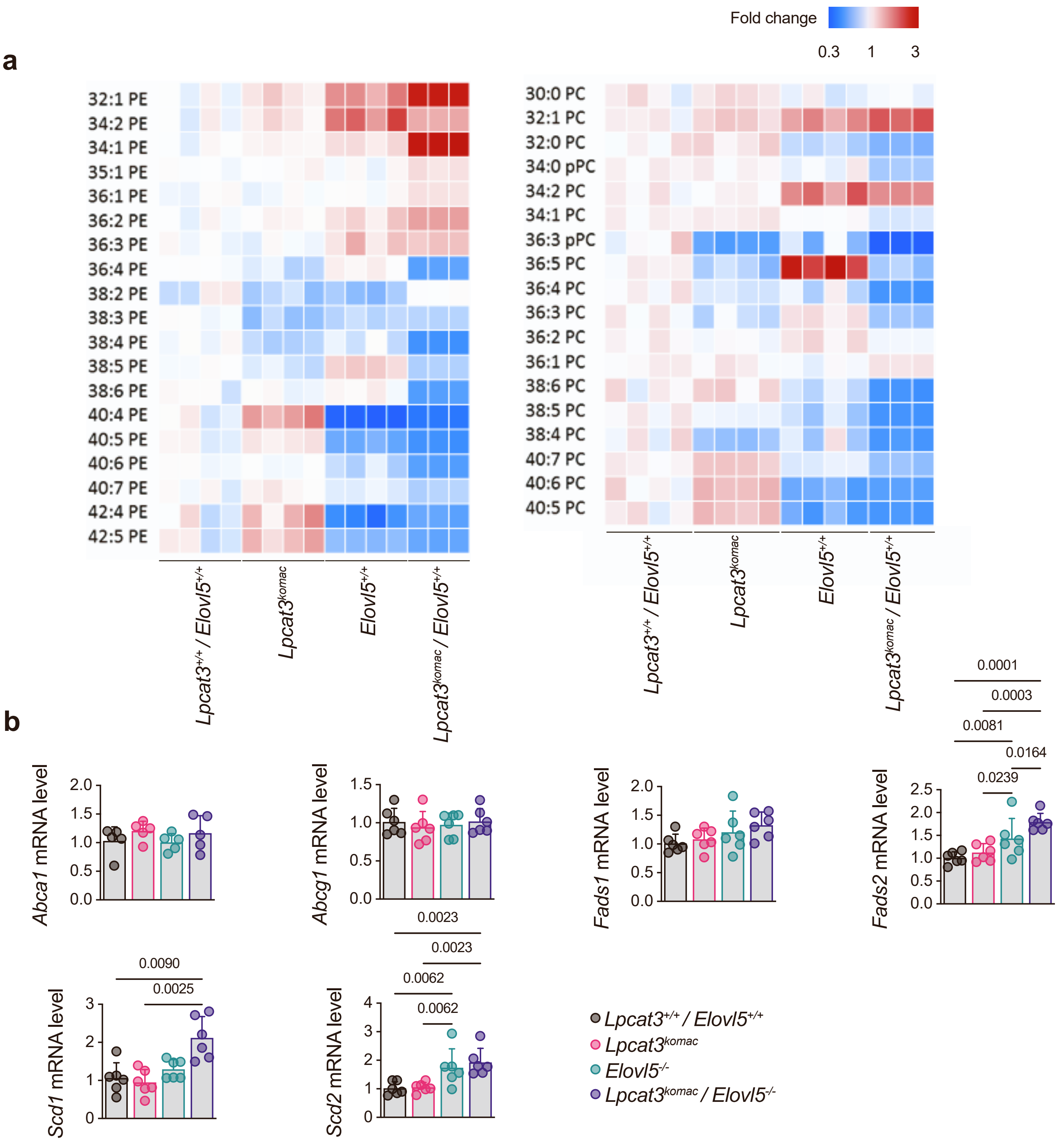

Figure S3. Relative mRNA levels of cholesterol transporters and major enzymes involved in unsaturated FA synthesis. *Related to Figure 2.*

(A) Heatmap of PEs and PCs in in *Lpcat3*$^{+/+}$/*Elovl5*$^{+/+}$, *Lpcat3*^{Komac}, *Elovl5*$^{-/-}$ and *Lpcat3*$^{Komac/}$*Elovl5*$^{-/-}$ primary macrophages. Data are expressed as percentage of total phospholipid subclass (*Lpcat3*$^{+/+}$/*Elovl5*$^{+/+}$, *Lpcat3*^{Komac}, *Elovl5*$^{-/-}$: n=4; *Lpcat3*^{Komac}/ *Elovl5*$^{-/-}$: n=3). (B). Relative mRNA levels of ABC transporters involved in cholesterol efflux and major enzymes involved in PUFA synthesis in *Lpcat3*$^{+/+}$/*Elovl5*$^{+/+}$, *Lpcat3*^{Komac,} *Elovl5*$^{-/-}$ and *Lpcat3*^{Komac}/*Elovl5*$^{-/-}$ primary macrophages normalized as 1 in *Lpcat3*$^{+/+/}$*Elovl5*$^{+/+}$ group (n=6). Values are mean ± SD. Statistical analysis performed with one-way ANOVA or Kruskal-Wallis test.

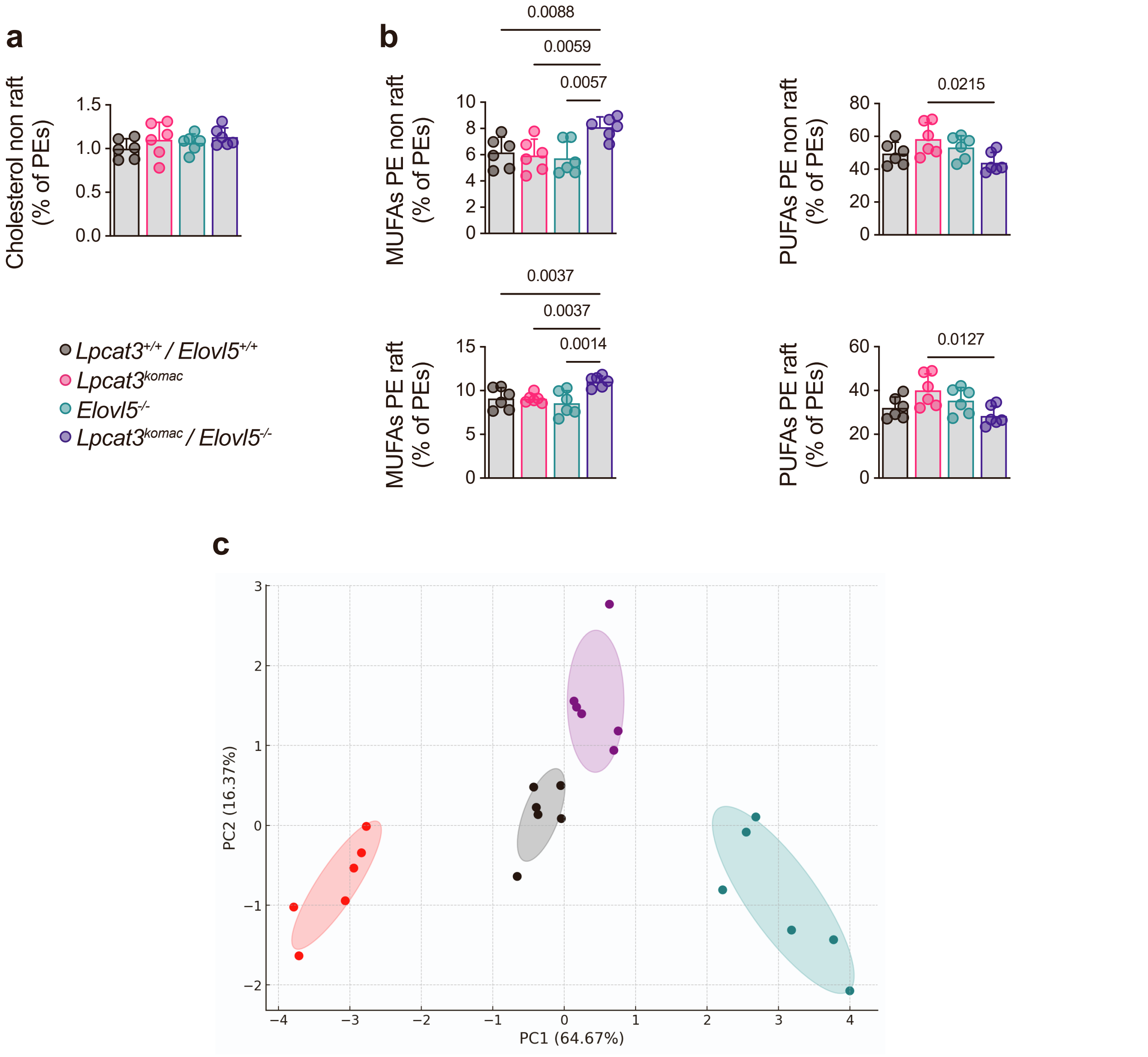

Figure S4. Lipid composition of DRMs and DSMs. *Related to Figure 3.* (A). Cholesterol content in heavy fractions from *Lpcat3*$^{+/+}$/*Elovl5*$^{+/+}$, *Lpcat3*^{Komac}, *Elovl5*$^{-/-}$ and *Lpcat3*^{Komac}/*Elovl5*$^{-/-}$ macrophages. Data are normalized at 1 in *Lpcat3*$^{+/+}$/*Elovl5*$^{+/+}$ group (n=6). (B). monounsaturated and polyunsaturated FA distribution in PEs in heavy and light fractions in *Lpcat3*$^{+/+}$/*Elovl5*$^{+/+}$, *Lpcat3*^{Komac}, *Elovl5*$^{-/-}$ and *Lpcat3*^{Komac}/*Elovl5*$^{-/-}$ macrophages. Data are expressed as percentage of PLs in light and heavy fractions (n=6). (C). Principal component analysis of phospholipid subclasses in DRMs from *Lpcat3*$^{+/+}$/*Elovl5*$^{+/+}$, *Lpcat3*^{Komac}, *Elovl5*$^{-/-}$ and *Lpcat3*^{Komac}/*Elovl5*$^{-/-}$ macrophages (n=6). Values are mean ± SD. Statistical analysis performed with one-way ANOVA.

a

	Leukocytes ($10^3/mm^3$)	Lymphocytes ($10^3/mm^3$)	Monocytes ($10^3/mm^3$)	Granulocytes ($10^3/mm^3$)	Eosinophils ($10^3/mm^3$)	Erythrocytes ($10^3/mm^3$)	Hemoglobin (g/dl)	Hematocrit (%)
Lpcat3$^{+/+}$ / *Elovl5*$^{+/+}$	8.81	4.96	0.33	3.51	0.78	8.82	14.95	44.6
Lpcat3^{komac}	12.75	7.14	0.4	3.81	1.32	8.20	14.7	42.1
Elovl5$^{-/-}$	12.33	8.02	0.4	3.83	0.98	8.26	15.48	44.5
Lpcat3^{komac} / *Elovl5*$^{-/-}$	8.97	6.12	0.31	2.53	0.37	9.48	16.05	47.7

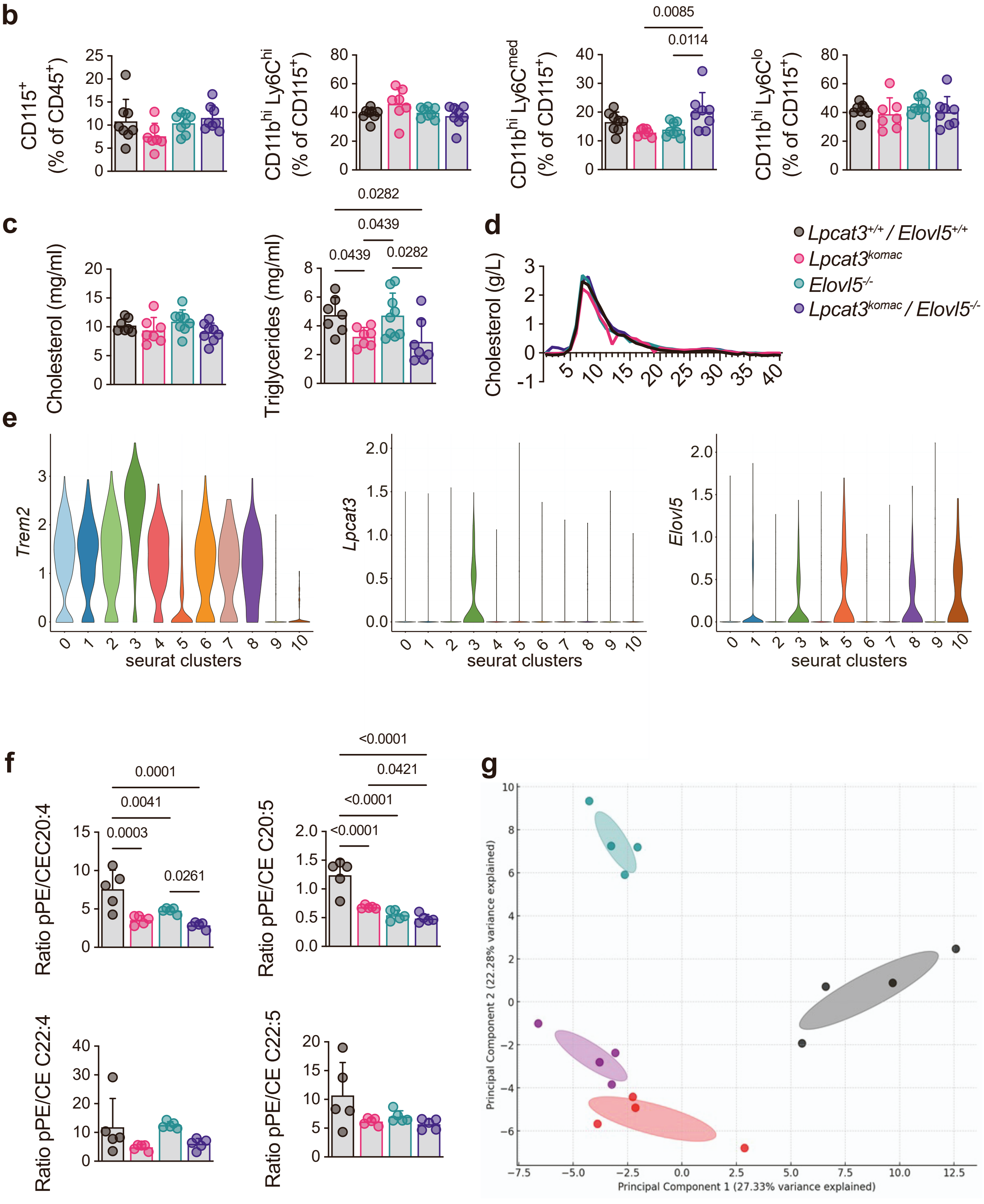

Figure S5. Hematological and Lipid parameters in Ldlr$^{-/-}$ mice transplanted with Lpcat3$^{+/+}$/Elovl5$^{+/+}$, Lpcat3^{Komac}, Elovl5$^{-/-}$ and Lpcat3^{Komac}/Elovl5$^{-/-}$ bone marrow under atherogenic diet. *Related to figure 4.*

(A). Hematological profile of *Ldlr*$^{-/-}$ mice transplanted with *Lpcat3*$^{+/+}$/*Elovl5*$^{+/+}$, *Lpcat3*^{Komac}, *Elovl5*$^{-/-}$ and *Lpcat3*^{Komac}/*Elovl5*$^{-/-}$ hematopoietic cells during the western type diet (n=8). (B). Percentage of CD115^{+}, CD11b^{hi}Ly6C^{hi}, CD11b^{hi}Ly6C^{med} and CD11b^{hi}Ly6C^{lo} amongst CD45^{+} assessed by flow cytometry of *Ldlr*$^{-/-}$ mice transplanted with *Lpcat3*$^{+/+}$/*Elovl5*$^{+/+}$, *Lpcat3*^{Komac}, *Elovl5*$^{-/-}$ and *Lpcat3*^{Komac}/ *Elovl5*$^{-/-}$ hematopoietic cells during the western type diet (n=8). (C-D). Plasma lipid parameters of *Ldlr*$^{-/-}$ mice transplanted with *Lpcat3*$^{+/+}$/*Elovl5*$^{+/+}$, *Lpcat3*^{Komac}, *Elovl5*$^{-/-}$ and *Lpcat3*^{Komac}/*Elovl5*$^{-/-}$ hematopoietic cells after twelve weeks of western type diet (n=8). (E). Expression of Trem2, Lpcat3 and Elovl5 in macrophages clusters from mouse atheroma plaques from public dataset (n=6 mice, 3781 CD45^{+} cells). (F). Ratio of PUFA enrichment between plasmalogen and cholesteryl esters in mouse atheroma plaques (n=5). (G). Principal component analysis of cholesteryl esters and phospholipid subclasses in atheroma plaques from *Ldlr*$^{-/-}$ mice transplanted with *Lpcat3*$^{+/+}$/*Elovl5*$^{+/+}$ and *Lpcat3*^{Komac}/ *Elovl5*$^{-/-}$ hematopoietic cells. Values are mean ± SD. Statistical analysis performed with one-way ANOVA. Correction for multiple comparison was performed by the procedure of Benjamini, Krieger and Yekutieli

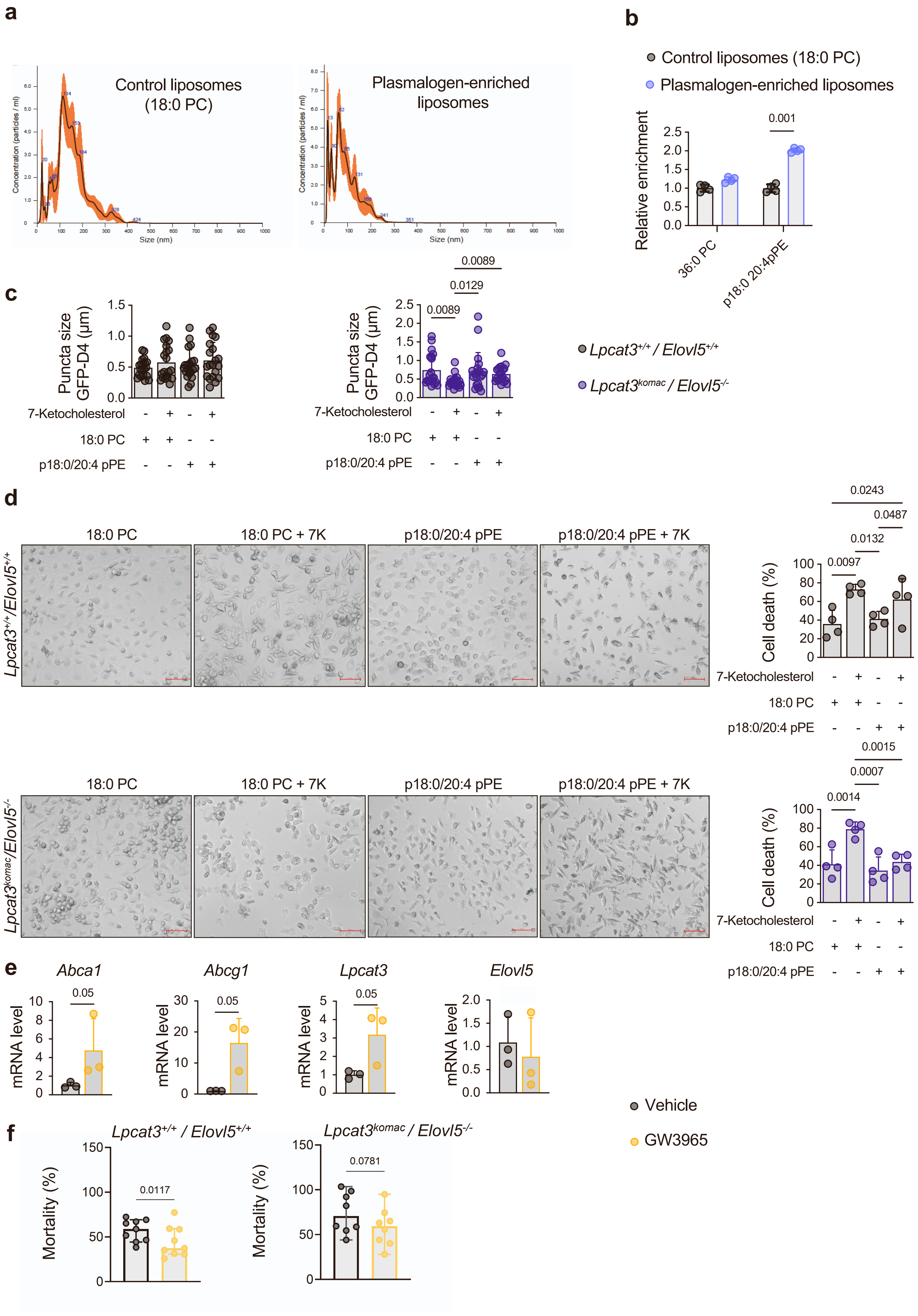

a
Control liposomes (18:0 PC)
Plasmalogen-enriched liposomes
Concentration (particles / ml)
Size (nm)
b
Control liposomes (18:0 PC)
Plasmalogen-enriched liposomes
Relative enrichment
0.001
36:0 PC
p18:0 20:4pPE
c
Puncta size GFP-D4 (µm)
7-Ketocholesterol
18:0 PC
p18:0/20:4 pPE
0.0089
0.0129
0.0089
Lpcat3+/+ / Elovl5+/+
Lpcat3komac / Elovl5-/-
d
18:0 PC
18:0 PC + 7K
p18:0/20:4 pPE
p18:0/20:4 pPE + 7K
Lpcat3+/+/Elovl5+/+
Lpcat3komac/Elovl5-/-
Cell death (%)
0.0243
0.0487
0.0132
0.0097
0.0015
0.0007
0.0014
e
Abca1
Abcg1
Lpcat3
Elovl5
mRNA level
0.05
0.05
0.05
Vehicle
GW3965
f
Lpcat3+/+ / Elovl5+/+
Lpcat3komac / Elovl5-/-
Mortality (%)
0.0117
0.0781

Figure S6. Impact of plasmalogen supplementation on mortality and cholesterol enriched domain formation in mouse BMDMs. *Related to Figure 5.*

(A). Particle size distribution for the Ctrl (18:0 PC) and plasmalogen (p18:0/20:4 pPE) enriched-liposomes. Measurements were obtained using a NanoSight system (NTA 3.4, Blue488), capturing particles in a size range from 0 to 1000 nm. Error bars indicate the standard error, representing statistical variability based on five independent measurements. (B). 18:0 PC and p18:0/20:4 pPE content in macrophages after incubation with liposomes. (C). Impact of 18:0 PC or p18:0/20:4 pPE supplementation on cholesterol enriched domains following 7-ketocholesterol treatment. (D). Impact of 18:0 PC or p18:0/20:4 pPE supplementation on cell death induced by 7-ketocholesterol treatment observed under optical microscope. % of necrotic and apoptotic cells were determined by analyzing macrophage morphology (n=4). (E). Analysis of the relative mRNA expression levels of *Abca1*, *Abcg1*, *Lpcat3*, and *Elovl5* under control (CTRL) and GW3965-treated conditions. (F). Assessment of mortality (%) in the *Lpcat3*$^{+/+}$/*Elovl5*$^{+/+}$ and *Lpcat3*^{Komac}/*Elovl5*$^{-/-}$ genotypes. Values are mean ± SD or median ± 95%CI. Statistical analysis performed with one-way ANOVA, Kruskal-Wallis test or Wilcoxon test for the impact of GW on mortality. Correction for multiple comparison was performed by the procedure of Benjamini, Krieger and Yekutieli

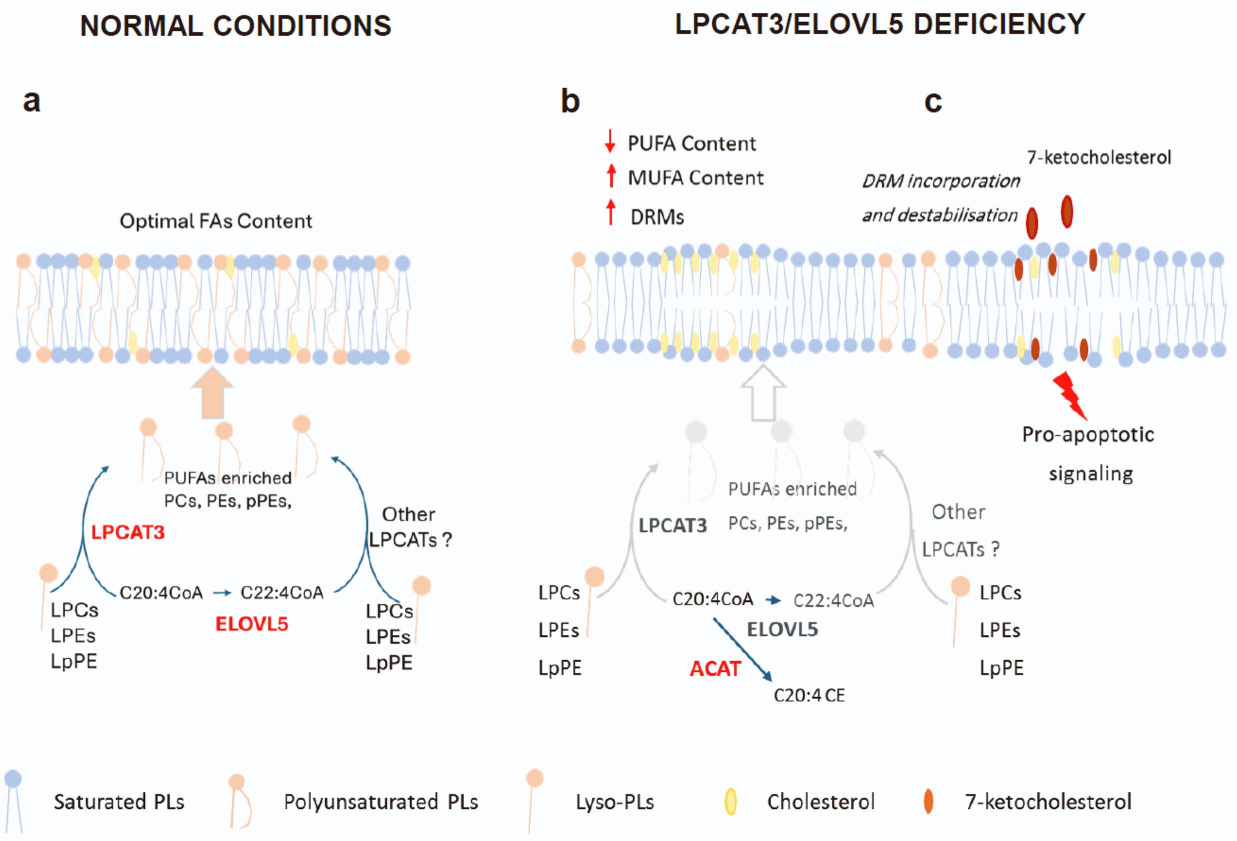

Figure S7. Role of LPCAT3 and ELOVL5 in membrane homeostasis. *Related to Figure 5.*

(A) Under normal conditions, the enzymes LPCAT3 and ELOVL5 work together to facilitate the efficient incorporation of polyunsaturated fatty acids (PUFAs) into the phospholipids of the cell membrane. LPCAT3 enables the acylation of lysophospholipids to form PUFA-enriched phospholipids, such as phosphatidylcholines (PCs), phosphatidylethanolamines (PEs), and plasmalogens (pPEs). ELOVL5 is involved in the elongation of fatty acids, contributing to the synthesis of longer fatty acid chains that can be incorporated by other LPCATs. (B) When both LPCAT3 and ELOVL5 are deficient, the lipid composition of the membrane is disrupted. The lack of LPCAT3 reduces PUFA incorporation into phospholipids, thereby lowering the levels of PUFA-enriched PCs, PEs, and pPEs. Without ELOVL5, the incorporation of longer fatty acids is also impaired. Unincorporated arachidonic acid is redirected toward the formation of cholesterol esters. This alteration leads to an increase in monounsaturated fatty acids (MUFAs) to compensate for the PUFA deficiency and results in increased cholesterol-enriched microdomains (DRMs).(C) Consequently, cells become more sensitive to cytotoxic oxysterols, which can incorporate into DRMs and destabilize them.

	Cluster 1 (n=81)	Cluster 2 (n=106)	p.overall
Age	73.0 [64.0;80.0]	72.5 [64.0;77.8]	0.479
Female	19 (23.5%)	28 (26.4%)	0.644
Type 2 diabetes	44 (54.3%)	49 (46.2%)	0.273
Symptomatic plaques	25 (30.9%)	47 (44.3%)	0.061
Presence of plaque ulcerations	10 (12.3%)	16 (15.1%)	0.590
Level of calcification:			<0.001
1	10 (12.3%)	20 (18.9%)	
2	48 (59.3%)	82 (77.4%)	
3	23 (28.4%)	4 (3.77%)	
Hypo-echogenic plaques	6 (7.41%)	25 (23.6%)	0.003
Hyper-echogenic plaques	35 (43.2%)	27 (25.5%)	0.011

Table S1. General characteristics of patients from cluster 1 and 2. *Related to figure 7.* Data are expressed as medians [25th -75th percentiles] or % of the population. Groups were compared with a student's test or Kruskal-Wallis non-parametric test; qualitative data were compared using chi-squared or Fischer's test.

Lipid Species	FC c2 vs c1	Adj P value
**Top lipid species cluster 1**		
**p18:0/20:4 pPE**	0.51	1.20E-13
**p18:0/22:4 pPE**	0.53	4.93E-13
**p18:0/16:0 pPE**	0.48	2.16E-12
**36:4 PC**	0.73	3.18E-12
**p18:0/22:6 pPE**	0.59	6.10E-09
**36:4 PE**	0.63	2.05E-08
**38:6 PE**	0.63	2.34E-08
**40:4 PE**	0.65	8.49E-08
**38:5 PC**	0.79	1.29E-07
**Top lipid species cluster 2**		
**34:0 pPC**	1.71	4.79E-21
**20:1 LPC lyso-2**	1.92	1.70E-13
**38:1 PC**	2.12	2.96E-12
**27 OH-Cholesterol**	4.93	1.55E-11
**20:2 LPC lyso-2**	1.69	8.88E-11
**32:1 PC**	1.38	2.96E-09
**7 alpha-OH-Cholesterol**	3.69	6.94E-09
**22:4 LPC lyso-2**	1.72	1.28E-08
**36:0 pPC**	1.89	1.26E-08
**7 keto-Cholesterol**	5.54	1.59E-08

Table S2. Top hits for lipid molecules enriched in clusters 1 and 2 respectively. *Related to Figure 7.* Data expressed as fold change between clusters. Groups were compared with a student's test or Mann-Whitney test and P values were adjusted by Benjamini-Hochberg procedure.

Murine primer	
Elovl5 sgRNA	CGGCAGCCGTTCTCTTGCCG
Gapdh	F : CAAGGTCATCCATGACAACTTTG<br>R : GGCCATCCACAGTCTTCTGG
Elovl5	F : GGTGTGTGGGAAGGCAAATA<br>R : AATTCGATGAGTTTGGAGAAGTAGTAC
Lpcat3	F : GGCCTCTCAATTGCTTATTTCA<br>R : AGCACGACACATAGCAAGGA
Abca1	F: CCGAGGAAGACGTGGACACCTTC<br>R : CCTCAGCCATGACCTGCCTTGTAG
Abcg1	F : ATGGGGTTGGTGCCAAAGA<br>R : AAACCTGGACAGGAAAGAATCC
Fads1	F : AGCACATGCCATACAACCATC<br>R : TTTCCGCTGAACCACAAAATAGA
Fads2	F : CTGGATGGCTGCAACATGACT<br>R : AGTTGGCTGAGGCACCCTTTA
Scd2	F : ATCTCTGGCGCTTACTCAGCC<br>R : GCCCCTCATCATCCTGATAGGT
Scd1	F : GGGTTGCCAGTTTCTTTCGT<br>R : GCCACAAAGCAAAGAAGGC

Table S3. Nucleotide sequences. *Related to Star Methods*
